# Supplementary material for: Pyfectious: An individual-level simulator to discover optimal containment policies for epidemic diseases
Source: PLoS Comput Biol. 2023 Jan 23;19(1):e1010799. doi: 10.1371/journal.pcbi.1010799 (PMC9894541; doi:10.1371/journal.pcbi.1010799)
Supplement: S1 File — This example is the best starting point to understand the simulator’s software interface and a comprehensive guide to run customized simulations with Pyfectious. A good knowledge of Python is necessary to invoke Pyfectious simulation capacity, especially if one decides to opt for manual simulation. (PDF) [file pcbi.1010799.s001.pdf]

S1 Manual Simulation

This example is the best place to understand the simulator's software interface and a comprehensive guide to designing and executing desired simulations with Pyfectious . Please follow each section and carefully read the instructions about customizing the simulation to fit any specific settings and requirements.

The code snippets presented in this section are already accessible on our comprehensive Google Colaboratory tutorials (`https://colab.research.google.com/drive/14UYB9x0g5s7jyHO4DynqDJMXTaSM11eW?usp=sharing`), where you can easily run the snippets one by one and get the results online, without any specific hardware or software requirements.

1 Import the required libraries

The source libraries have to be included in the environment to start a simulation.

```
import sys, os

# All the required lib files are located in the source folder
sys.path.insert(1, os.path.join(os.pardir, 'src'))
```

2 Build a test environment

This section starts from scratch and builds up all the necessary elements of a simulation environment. Moreover, these settings can also be saved in JSON format for later use cases. In the following steps, we demonstrate the process of building a simple simulation configuration.

Note that this process is just for understanding the fundamental software concepts; therefore, in reality, there is no need to start from scratch, and one can use the tutorial presented in S2 Configured simulation to run their customized simulation.

2.1 Family patterns dictionary

The family pattern is the first object required to build the population generator class. Below is the procedure to construct a family pattern dictionary. The family pattern dictionary resembles the general pattern of the families in the simulation.

Location Creating a sample location distribution can be as easy as importing the Test module. Alternatively, customized distributions may be developed with the help of modules in distributions.py.

```
from population_generator import Test
location_distribution = Test.get_location_distribution()
```

Age Age distributions can be created using the distribution classes implemented in distributions.py, like the following code snippet. Accordingly, build the age distributions list to gather all the distributions in one place. A more advanced distribution can be developed using the interface provided in the distributions.py.

```
from distributions import Truncated_Normal_Distribution

# Adults age distribution
age_distribution_2 = Truncated_Normal_Distribution({"lower_bound": 30,
    "upper_bound": 50, "mean": 40, "std": 5})
age_distribution_1 = Truncated_Normal_Distribution({"lower_bound": 20,
    "upper_bound": 40, "mean": 30, "std": 5})

# Children age distribution
age_distribution_3 = Truncated_Normal_Distribution({"lower_bound": 5,
    "upper_bound": 15, "mean": 10, "std": 3})
age_distribution_4 = Truncated_Normal_Distribution({"lower_bound": 0,
    "upper_bound": 5, "mean": 2.5, "std": 2})

age_distributions = [age_distribution_1, age_distribution_2]
```

Health condition Health condition distribution is more or less determined in the same way as age distribution. However, a person's health condition is modeled by a number between 0 and 1, where one means the person has no history of significant health problems.

```
# Normal distribution is used here to describe the population's health
    condition
health_condition_distribution_1 =
    Truncated_Normal_Distribution({"lower_bound": 0, "upper_bound": 1,
                                                          "mean": 0.5,
                                                              "std":
                                                              0.1})
health_condition_distribution_2 =
    Truncated_Normal_Distribution({"lower_bound": 0, "upper_bound": 1,
                                                          "mean": 0.5,
                                                              "std":
                                                              0.1})
# A list of all the distributions must be assembled
```

```
health_condition_distributions = [health_condition_distribution_1,
    health_condition_distribution_2]
```

Family pattern Now we have almost all the required fields to generate a family pattern. We also need to create a gender list consisting of all the family members, respectively. Two instances of creating a family pattern are mentioned in the following code snippet to demonstrate the object's flexibility in modeling any patterns.

```
# Size of the family
number_of_members = 2

# Create a genders list based on the number of members
genders = [0, 1]

# Build the family pattern object
from population_generator import Family_Pattern
family_pattern_1 = Family_Pattern(number_of_members,
                                  age_distributions,
                                  health_condition_distributions,
                                  genders,
                                  location_distribution)

# Family size is increased here
number_of_members = 4

# For a four member family, age and health condition distributions most
    be of length 4, respectively
age_distributions = [age_distribution_1, age_distribution_2,
    age_distribution_3, age_distribution_4]

health_condition_distributions = [health_condition_distribution_1,
    health_condition_distribution_2, health_condition_distribution_1,
    health_condition_distribution_2]

# Here we have male and female equally
genders = [0, 1, 1, 0]

# Build the family pattern object
family_pattern_2 = Family_Pattern(4, age_distributions,
    health_condition_distributions, genders, location_distribution)
```

Probability dictionary Last but not least, the job here is to build a family probability dictionary. This structure represents the presence probability of each family pattern in society. Naturally, the accumulative sum of the probabilities must be equal to 1.

```
# note that the sum of probabilities must be 1
family_pattern_probability_dict = {family_pattern_1: 0.4,
    family_pattern_2: 0.6}
```

2.2 Community types

A community type object represents the overall structure of a specific community, e.g., a school, in the simulation environment. Each community type consists of a list of sub-community types, for instance, teacher, student, etc., and a sub-community connectivity dictionary, representing the interactions between sub-communities as a graph. Name and location distribution are also other parts of the structure.

Sub-community types Sub-community types represent a smaller community, generally attached to a particular community type role, e.g., student and teachers. To build a sub-community type, the procedure indicated in the following code snippet must be followed.

```
# Generate an age distribution for this sub-community
age_distribution = Truncated_Normal_Distribution({"lower_bound": 5,
    "upper_bound": 15, "mean": 10, "std": 1})

# Create a time cycle distribution, when people are present in this
    community
from distributions import Uniform_Whole_Week_Time_Cycle_Distribution
time_cycle_distribution = Uniform_Whole_Week_Time_Cycle_Distribution({
    "start": {
        "lower_bound": 300,
        "upper_bound": 301
    },
    "length": {
        "lower_bound": 250,
        "upper_bound": 500
    }
})

# Generate a community type role object
from population_generator import Community_Type_Role
student_type = Community_Type_Role(age_distribution,
                                   Test.get_gender_distribution(),
                                   time_cycle_distribution,
                                   True,
                                   1)

# Build the number of members distribution
from distributions import UniformSet_Distribution
number_of_members_distribution = UniformSet_Distribution({
```

```
    "probability_dict": {
        30: 1,
    }
})

# Generate the sub community type using the Role, members and a
    connectivity distribution
from population_generator import Sub_Community_Type
sub_community_type_1 = Sub_Community_Type(student_type,
"Student",
                    number_of_members_distribution,
                    Test.get_connectivity_distribution(),
                    Test.get_transmission_potential_distribution())
```

Another sub-community type is generated below.

```
age_distribution = Truncated_Normal_Distribution({"lower_bound": 20,
    "upper_bound": 40, "mean": 30, "std": 5})
teacher_type = Community_Type_Role(age_distribution,
                               Test.get_gender_distribution(),
                               time_cycle_distribution,
                               True,
                               1)
number_of_members_distribution = UniformSet_Distribution({
    "probability_dict": {
        5: 1,
    }
})
sub_community_type_2 = Sub_Community_Type(teacher_type, "Teacher",
                    number_of_members_distribution,
                    Test.get_connectivity_distribution(),
                    Test.get_transmission_potential_distribution())
```

Build a community type The community type object can now be created by putting together the sub-community types and connectivity distributions. It is noteworthy that connectivity dictionary explains the level of mutual contacts between sub-communities by employing a probabilistic distribution. Here, we use a prepared distribution that has already been implemented in the Test class.

The transmission potential explains the possibility of virus transmission between the individuals of a community. For instance, infectious diseases can spread more quickly in a closed environment such as a classroom.

```
from population_generator import Community_Type

# Build the community types list
subcommunity_types_list = [sub_community_type_1, sub_community_type_2]
```

```
# Build the connectivity dictionary for sub-communities
sub_community_connectivity_dict =
    {0:{1:Test.get_connectivity_distribution()},
   1:{0:Test.get_connectivity_distribution()}}
# Prepare a transmission potential dictionary
transmission_potential_dict =
              {0: {1:Test.get_transmission_potential_distribution()},
               1: {0:Test.get_transmission_potential_distribution()}}

# Build the community type object
school_type = Community_Type(sub_community_types=subcommunity_types_list,
       name="School",
       number_of_communities=4,
       sub_community_connectivity_dict=sub_community_connectivity_dict,
       location_distribution=Test.get_location_distribution(),
       transmission_potential_dict=transmission_potential_dict)
```

2.3 Population generator

Having the family pattern dictionary and community types, a population generator may be created in the following way. This class contains all the necessary information to generate a sample population in the simulation environment.

```
from population_generator import Population_Generator

# Build the Population Generator class
population_generator = Population_Generator(population_size=500,
       family_pattern_probability_dict=family_pattern_probability_dict,
       community_types=[school_type, school_type])
```

Moreover, the following command examines how the population is spread among the families and communities. More importantly, The generate_population method is necessary in order to build an operational set of parameters later used during the simulation procedure. For large populations, it is also possible to run this task using the python multiprocessing library by just setting is_parallel to True.

```
people, graph, families, communities =
    population_generator.generate_population(is_parallel=False)
```

2.4 Disease properties

The disease properties class represents the major specifications related to the spread of the target infectious disease. Any disease specifications may be applied here, e.g., attributes related to COVID-19, MERS, and SARS behavior.

```python
from distributions import Uniform_Disease_Property_Distribution
from time_handle import Time

# infection rate
infection_rate_distribution = Uniform_Disease_Property_Distribution({
    "lower_bound": 0.6,
    "upper_bound": 0.8
})

# Immunity rate
immunity_distribution = Uniform_Disease_Property_Distribution({
    "lower_bound": 0.05,
    "upper_bound": 0.15
})

# Disease period
disease_period_distribution = Uniform_Disease_Property_Distribution({
    "lower_bound": Time.convert_day_to_minutes(2),
    "upper_bound": Time.convert_day_to_minutes(5)
})

# Incubation period
incubation_period_distribution = Uniform_Disease_Property_Distribution({
    "lower_bound": Time.convert_day_to_minutes(7),
    "upper_bound": Time.convert_day_to_minutes(16)
})

# Death probability
death_probability_distribution = Uniform_Disease_Property_Distribution({
    "lower_bound": 0.05,
    "upper_bound": 0.1
})

from disease_manipulator import Disease_Properties

# Creating the disease properties object
disease_properties = Disease_Properties(
        infection_rate_distribution=infection_rate_distribution,
        immunity_distribution=immunity_distribution,
        disease_period_distribution=disease_period_distribution,
        death_probability_distribution=death_probability_distribution,
        incubation_period_distribution=incubation_period_distribution)
```

3 Deploying the simulation

Now we start working with the simulator class. The upcoming sections will illustrate the entire simulator's execution process.

3.1 Primary settings

The simulator starts with the population generator and disease properties objects as base settings. Afterward, the generate_model function steps in to generate a simulation environment, such as people, families, and communities, as well as preparing the ground for simulating in the following steps.

```
from time_simulator import Simulator

# Instantiate the simulator object using previously prepared population
    generator and disease properties objects
simulator = Simulator(population_generator, disease_properties)

# Execute the model generation method
simulator.generate_model()
```

End time The simulation end time is crucial since it determines how long the simulation should keep going. Here we set a 60-day simulation, starting from now.

```
from datetime import datetime, timedelta
from time_handle import Time

end_time = Time(delta_time=timedelta(days=60),
    init_date_time=datetime.now())
```

Spread period Determining the spread period is crucial since it clarifies the simulation's granularity. To have a detailed simulation, the user must set lower values where the spread sequence is investigated frequently. Otherwise, increasing the virus spread period causes a reduction in computational costs.

```
spread_period = Time(delta_time=timedelta(hours=1))
```

Initially infected people Any pandemic must start from certain people, i.e., the initially infected subjects. A list of id numbers represents the initially infected people.

```
import random

initial_infected_ids = random.sample(range(500), 10)
```

Observers The observer is the module responsible for saving data into the database. Using the observer, the data during the simulation can be stored

and later be used in plots, reasoning, etc. The simulator can handle a list of observers with various trigger conditions.

```
from observer import Observer
from conditions import Time_Period_Condition

# Build the observer object
observer =
    Observer(Time_Period_Condition(Time(delta_time=timedelta(hours=5))),
    True)

# Generate the observers list
observers_list = [observer]
```

Commands The command list is used to create a policy to contract the pandemic. A simple strict command may be to quarantine all the communities. At this point, we leave the list empty to run a simple simulation.

```
commands_list = []
```

3.2 Simulate

At this stage, the only remaining step is running the simulation. This might take a while, depending on the population size and the total end time. Other factors, such as the number of observers, are also influential in determining the simulation time.

The report statistics can be varied from 0 (default) to 1 and 2 if there is a need of reviewing more details of the simulation at the end.

```
# Execute the simulation using the simulate method
simulator.simulate(end_time=end_time,
                   spread_period=spread_period,
                   initialized_infected_ids=initial_infected_ids,
                   commands=commands_list,
                   observers=observers_list,
                   report_statistics=2)
```

4 Plot the results

Here are some useful plots obtained by utilizing the observer's methods to evaluate and analyze the simulation's results. The data associated with a specific observer may be retrieved using the simulator.database functions. However, the observer object provides some useful plots and automatic data derivation

without any need to be directly connected to the simulation database. The plots associated with the following code snippet are presented in Figure 1.

```
observer.plot_initial_bar_gender()
observer.plot_initial_hist_age()
observer.plot_initial_hist_health_condition()

from utils import Health_Condition, Infection_Status
observer.plot_disease_statistics_during_time(Health_Condition.DEAD)
observer.plot_final_hist_age(Health_Condition.HAS_BEEN_INFECTED)
observer.plot_disease_statistics_during_time(Health_Condition.IS_INFECTED)
```

5 Add a condition

A new condition can be designed and developed using the following structure and by inheriting the Condition class. The is_satisfied function determines whether the condition is satisfied and is_able_to_be_removed determines whether the condition is useless from now on or not.

```
from conditions import Condition

# Build your own customized condition
class More_Than_X_Deaths_Condition(Condition):

    def __init__(self, x):
        super().__init__()
        self.x = x
        self.satisfied = False

    def is_satisfied(self, simulator: Simulator, end_time: Time):
        temp = self.x
        for person in simulator.people:
            if not person.is_alive:
                temp -= 1
        if temp <= 0:
            self.satisfied = True
            return [simulator.clock]
        return []

    def is_able_to_be_removed(self):
        return self.satisfied
```

The newly generated condition may be used in both observers and commands. Here is an example of how to use the condition in an observer.

```
from observer import Observer
observer = Observer(More_Than_X_Deaths_Condition(10), True)
```

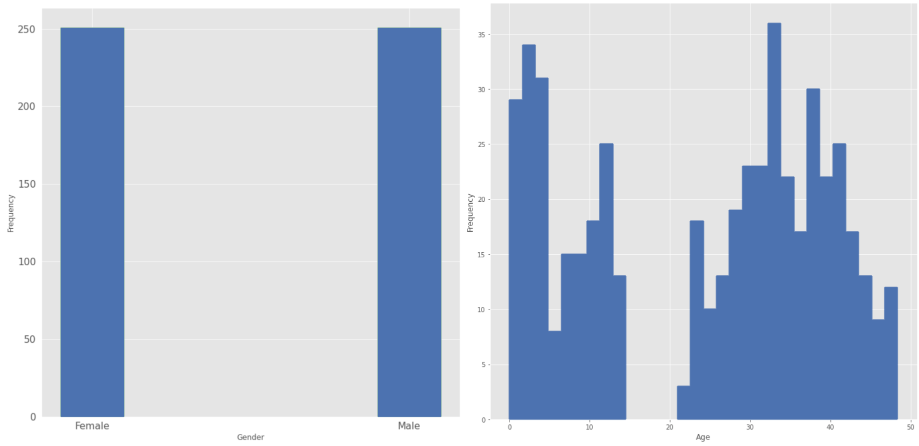

(a) Gender and age distribution of the simulator's population

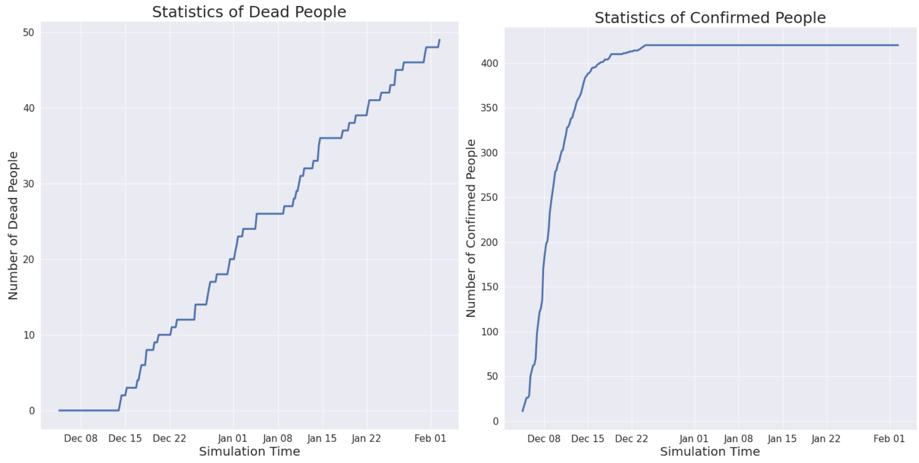

(b) Statistics of dead and confirmed cases over time

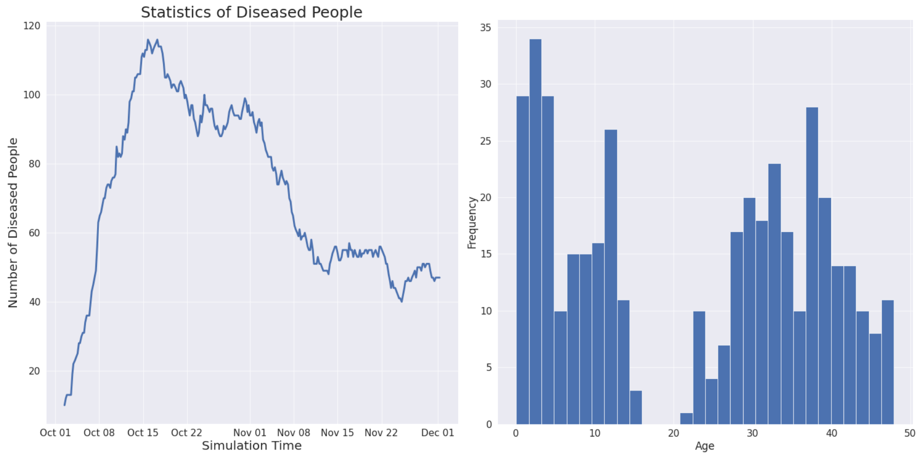

(c) Statistics of active cases and frequency of confirmed cases at the end of the simulation time

Figure 1: The results of observer plots

```
# Generate the observers' list
observers_list = [observer]

simulator.simulate(end_time=end_time,
                   spread_period=spread_period,
                   initialized_infected_ids=initial_infected_ids,
                   commands=commands_list,
                   observers=observers_list)
```

6 Add a command

Similarly, as adding a condition, a developer can also add a command using the Command class. New commands should follow the base class structure and functions in order to work correctly. The action that a particular command is supposed to take can be specified in the take_action method.

```
from commands import Command

# Build your own customized command
class Quarantine_Diseased_People(Command):
    def __init__(self, condition: Condition):
        super().__init__(condition)
        self.condition = condition

    def take_action(self, simulator: Simulator, end_time: Time):
        if self.condition.is_satisfied(simulator, end_time):
            for person in simulator.people:
                if person.infection_status is Infection_Status.CONTAGIOUS
                    or \
                       person.infection_status is
                            Infection_Status.INCUBATION:
                   person.quarantine()

    # This method is necessary due to automation purposes
    def to_json(self):
        return dict(name=self.__class__.__name__,
                   condition=self.condition)
```

7 Save the main objects as JSON configuration files

The objects can be saved as JSON files. These files may also be employed later to avoid preparations.

```python
from json_handle import Parser

# Build parser object
json_parser = Parser()

# Save population generator
json_parser.build_json(population_generator)
json_parser.save_json()

# Save disease properties
json_parser.build_json(disease_properties)
json_parser.save_json()

# Save population generator
json_parser.build_json(simulator)
json_parser.save_json()
```
