# Supplementary material for: Pyfectious: An individual-level simulator to discover optimal containment policies for epidemic diseases
Source: PLoS Comput Biol. 2023 Jan 23;19(1):e1010799. doi: 10.1371/journal.pcbi.1010799 (PMC9894541; doi:10.1371/journal.pcbi.1010799)
Supplement: S2 File — This file is dedicated to explaining the automated configuration process of the simulator. As opposed to manually building the population structure, quarantine strategies, and disease models, the data required by the simulator is obtained from hierarchical JSON configuration files. Using JSON configuration files makes it easier for people with limited knowledge of python, or programming in general, to interact with the software. (PDF) [file pcbi.1010799.s002.pdf]

S2 Configured Simulation

This section is dedicated to explaining the automated setup procedure of the simulator. As opposed to manually building the population and disease models, the data required by the simulator is obtained from already prepared JSON configuration files.

The code snippets presented in this section are already accessible on our comprehensive Google Colaboratory tutorials (`https://colab.research.google.com/drive/14UYB9x0g5s7jyHO4DynqDJMXTaSM11eW?usp=sharing`), where you can easily run the snippets one by one and get the results online, without any specific hardware or software requirements.

1 Create a settings folder

A folder called 'data', located in the project's main directory, consists of four major parts: json, figure, pickle, and sql. The sql folder, as appears of its name, is related to the simulation database, and the database files are stored there. The json folder is used to prepare the simulator's configuration files. By opening the json folder, some samples are already designed and placed there under the respective folders. These are some experimental configuration files that may be used to run a simulation.

For instance, open the folder named 'test'. Under this folder, there are three JSON files as explained below.

1. Population_Generator.json: This file consists of the information required to build an entire population generator object.
2. Disease_Properties.json: This file consists of the information required to build an entire disease properties object. Parameters like the infection rate and immunity are sub-fields of this JSON file.
3. Simulator.json: This file consists of the data required to call the simulate function in the Simulator class, including end_time, spread_period, commands, etc.

1.1 Customized configuration files

To build a customized setting, the folder named 'test' must be copied and pasted as a new folder, and name it as you like. For instance, here, we create a copy

and call it ‘configured_test’. Afterward, it is possible to change the JSON files’ values to fit other criteria such as larger population, other diseases, and more complex population structures.

```
import os
import sys
sys.path.insert(1, os.path.join(os.pardir, 'src'))

# Check if we are in 'test' folder right now
print(f'Current directory: {os.getcwd()}')

# Change directory to 'data' and then to 'json' folder
os.chdir(os.path.join(os.pardir, 'data', 'json'))
```

```
# Build the configure_test folder if does not exists
try:
    os.mkdir('configured_test')
except FileExistsError:
    pass

# Determine source and destination for copy operation
destination = os.path.join(os.getcwd(), 'configured_test')
source = os.path.join(os.getcwd(), 'test')

# Copy the items in 'test' to 'configured_test'
import shutil
src_files = os.listdir(source)
for file_name in src_files:
    full_file_name = os.path.join(source, file_name)
    if os.path.isfile(full_file_name):
        shutil.copy(full_file_name, destination)
```

Now, we can try to make some changes to the JSON configuration files. For the sake of simplicity, we change the population size to 800. This can be done either manually, from an editor, or using a script like the following.

```
# Considering the last section, we are now in the 'json' folder, so we
    move to the 'data' folder again so that the configured test is
    accessible.
os.chdir(os.pardir)

# Import and initialize the parser
from json_handle import Parser
parser = Parser(folder_name='configured_test')

# Parse the Population_Generator.json in 'configured_test'
population_generator = parser.parse_population_generator()
print(f'Population size before the change is:
```

```
    {population_generator.population_size}')

# Change the population size
population_generator.population_size = 800

# Save the new population generator as json
parser.build_json(population_generator)
parser.save_json()

# Parse the Population_Generator.json in 'configured_test'
population_generator = parser.parse_population_generator()
print(f'Population size after the change is:
    {population_generator.population_size}')
```

2 Simulation

In this section, we simulate data based on the settings saved inside the 'configured_test' folder.

```
# Import and initialize the parser
from json_handle import Parser
parser = Parser(folder_name='configured_test')


# Load Simulator from JSON file
simulator = parser.parse_simulator()
simulator.generate_model()

# Check the population size after generation
print(f'Population size is: {len(simulator.people)}')
```

Additionally, the policy and simulation specifics must be obtained from the Simulator.json as described below.

```
# Load Simulator Data from JSON file
end_time, spread_period, initialized_infected_ids, commands, observers =
    parser.parse_simulator_data()

# Run the simulation
simulator.simulate(end_time=end_time,
                   spread_period=spread_period,
                   initialized_infected_ids=initialized_infected_ids,
                   commands=commands,
                   observers=observers)
```

We have completed the simulation, and the results may be obtained from the database or statistics.

```
from utils import Health_Condition
observers[0].plot_disease_statistics_during_time(Health_Condition.IS_INFECTED)
```

Finally, having access to a summary of the simulation is possibly either by setting the report_statistics option of the simulate function, or separately calling in the statistics class.

```
simulator.statistics.show_people_statistics(simulator=simulator)
```

```
INFO - utils.py - 303 - show_people_statistics - 2020-12-04 12:58:56,722
    -
+----------------------------+-------+
|           People           | Count |
+============================+=======+
| Population Size            | 800   |
+----------------------------+-------+
| Confirmed (Active + Close) | 150 |
+----------------------------+-------+
| Total Death Cases          | 5     |
+----------------------------+-------+
| Total Recovered            | 795   |
+----------------------------+-------+
| Currently Active Cases     | 0     |
+----------------------------+-------+
```

3 The example town

In this section, our sample town, implemented under the 'json' folder with 50k population size, six family patterns, and major communities, including schools and workplaces, gyms, and restaurants, is being tested.

3.1 Parse the configuration files

Prior to anything else, we have to parse the configuration files located in the town folder, under data/json directory.

```
# Import libs
import sys, time, os

sys.path.insert(1, os.path.join(os.pardir, 'src'))

# Import and initialize the parser
from json_handle import Parser
parser = Parser(folder_name='town')
```

```
# Load Simulator from JSON file
simulator = parser.parse_simulator()
```

3.2 Generate and save the model

Since the model obtained by the generate_model function in this simulation is often large, we can utilize the simulator power to save the model for later use by employing the simulator.save_model method, and later use it using the simulator.load_model method.

```
# Generate the simulation model
simulator.generate_model()

# Time the generation process
end_generate_model = time.time()

# Save the simulation model
simulator.save_model('town')

# Save the simulation model
simulator.load_model('town')
```

3.3 Simulate the town

After the model is generated, we simulate the town in this section.

```
# Load Simulator Data from JSON file
end_time, spread_period, initialized_infected_ids, commands, observers =
    parser.parse_simulator_data()

# Run the simulation
simulator.simulate(end_time=end_time,
                   spread_period=spread_period,
                   initialized_infected_ids=initialized_infected_ids,
                   commands=commands,
                   observers=observers,
                   report_statistics=2)
```

3.4 Evaluate the results

In the end, we present some plots in Figure 1 to illustrate the simulation's results.

```
observers[0].plot_disease_statistics_during_time(Health_Condition.IS_INFECTED)

observers[0].plot_disease_statistics_during_time(Health_Condition.DEAD)
```

```
observers[0].plot_disease_statistics_during_time(Health_Condition.HAS_BEEN_INFECTED)

observers[0].plot_disease_statistics_during_time(Health_Condition.IS_NOT_INFECTED)
```

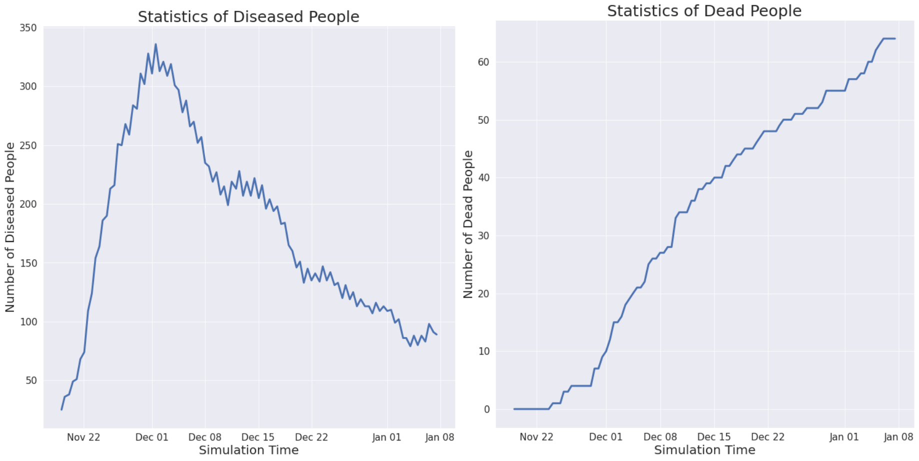

(a) Statistics of dead and infected people over time

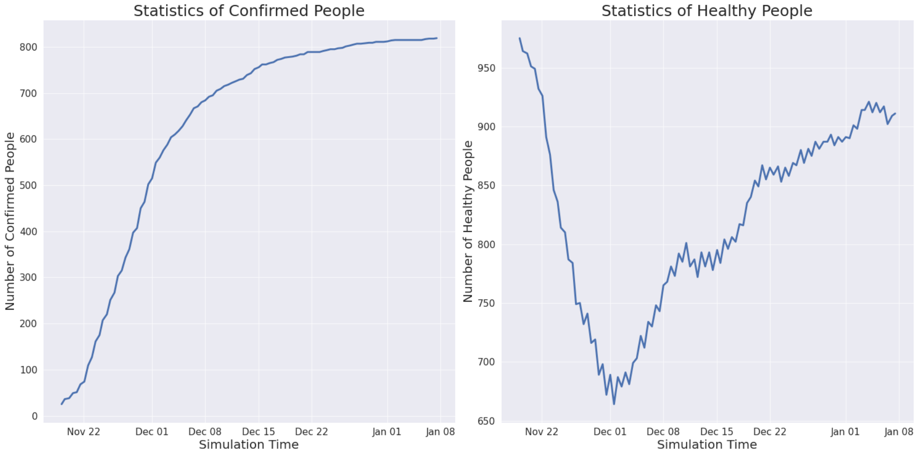

(b) Statistics of healthy and confirmed cases over time

Figure 1: The results of observer plots
