# Supplementary material for: Pyfectious: An individual-level simulator to discover optimal containment policies for epidemic diseases
Source: PLoS Comput Biol. 2023 Jan 23;19(1):e1010799. doi: 10.1371/journal.pcbi.1010799 (PMC9894541; doi:10.1371/journal.pcbi.1010799)
Supplement: S3 File — We demonstrate the procedure of conducting further experiments in order to evaluate the basic functionality and sanity of the simulator. Prior to engaging with this tutorial, one should take a look at S1 File, explaining the manual simulation process. (PDF) [file pcbi.1010799.s003.pdf]

S3 Sanity Checks

In this section, we demonstrate the procedure of conducting further experiments in order to evaluate the basic functionality and sanity of the simulator. Prior to engaging with this part, one should take a look at Manual Simulation.

1 Import the necessary libraries

In the beginning, we import some necessary simulation libraries from the code folder.

```
import sys, os
sys.path.insert(1, os.path.join(os.pardir, 'src'))
```

2 Run a normal simulation

This is the most basic form of the simulation, with no commands or, in other words, no applied policies. In the first step, we initialize the parser and load population generator and disease properties configuration files from the individual JSON files.

```
# Import Parser
from json_handle import Parser
parser = Parser('test')

# Load Population Generator from JSON file
population_generator = parser.parse_population_generator()

# Load Disease Properties from JSON file
disease_properties = parser.parse_disease_properties()
```

Then, simulator settings are parsed and loaded into the simulator. This also includes the last two steps, so there is no need for the previous code snippet, and it is generally used for explanation or debug purposes.

```
# Load the simulator from JSON file
simulator = parser.parse_simulator()
simulator.generate_model()
```

Now we load simulator data as well.

```
# Load simulator's data from JSON file
end_time, spread_period, initialized_infected_ids, _, observers =
    parser.parse_simulator_data()
```

We set the commands to an empty list and run the simulation.

```
# Run the simulation
simulator.simulate(end_time=end_time,
                  spread_period=spread_period,
                  initialized_infected_ids=initialized_infected_ids,
                  commands=[], # No commands needed at this time

                  observers=observers)
```

To observe the simulation results, we plot the curve of the active cases using the observer object. The result appears in Figure 1a.

```
from utils import Health_Condition
observers[0].plot_disease_statistics_during_time(Health_Condition.IS_INFECTED)
```

3 Quarantine everyone

In the next step, we add a policy to quarantine all the people after 20 days, a trivial form of quarantine, and see how the results change. You can compare the results from this section and section 3 in Figure 1a.

```
# Import Parser
from json_handle import Parser
parser = Parser('test')

# Load the simulator from JSON file
simulator = parser.parse_simulator()
simulator.generate_model()

# Load simulator's data from JSON file
end_time, spread_period, initialized_infected_ids, _, observers =
    parser.parse_simulator_data()

# Build a general quarantine policy
from datetime import timedelta
from commands import Quarantine_Multiple_People
from conditions import Time_Point_Condition

commands = [Quarantine_Multiple_People(
                  condition=Time_Point_Condition(Time(timedelta(days=20))),
```

```
                  ids=[i for i in range(500)])]

# Execute the simulation
simulator.simulate(end_time,
                  spread_period,
                  initialized_infected_ids,
                  commands,
                  observers,
                  report_statistics=2)

# Plot the results
observers[0].plot_disease_statistics_during_time(Health_Condition.IS_INFECTED)
```

4 Quarantine infected people

A more logical form of quarantine is to quarantine only the infected people at some point during the simulation, e.g., day 15, and naturally the results, presented in Figure 1b, should be the same as Section 3 since the people who are not infected do not pose any threats in case they are not quarantined.

```
# Import Parser
from json_handle import Parser
parser = Parser('test')

# Load the simulator from JSON file
simulator = parser.parse_simulator()
simulator.generate_model()

# Load simulator's data from JSON file
end_time, spread_period, initialized_infected_ids, _, observers =
    parser.parse_simulator_data()

# Build a policy
from datetime import timedelta
from commands import Quarantine_Diseased_People
from conditions import Time_Point_Condition
commands = [Quarantine_Diseased_People(
                  condition=Time_Point_Condition(Time(timedelta(days=15))))]

# Execute the simulation
simulator.simulate(end_time,
                  spread_period,
                  initialized_infected_ids,
                  commands,
                  observers,
                  report_statistics=2)
```

```
# Plot the results
from utils import Health_Condition
observers[0].plot_disease_statistics_during_time(Health_Condition.IS_INFECTED)
```

5 Infection rate

Here, we set the infection rate to a meager amount and check the test result. After setting the infection rate to another higher value, we compare the two curves in Figure 1b.

```
# Import Parser (default constructor is the 'test' folder)
from json_handle import Parser
parser = Parser('test')

# Load the simulator from JSON file
simulator = parser.parse_simulator()

# Change the infection rate here (or just change in the respective JSON
    file and then parse the simulator)
from distributions import Uniform_Disease_Property_Distribution
simulator.disease_properties.infectious_rate_distribution = \
   Uniform_Disease_Property_Distribution(parameters_dict={"upper_bound":0.2,
       "lower_bound":0.1})

# Generate the simulation model
simulator.generate_model()

# Load simulator's data from JSON file
end_time, spread_period, initialized_infected_ids, _, observers =
    parser.parse_simulator_data()

# No policy is required
commands = []

# Execute the simulation
simulator.simulate(end_time,
                  spread_period,
                  initialized_infected_ids,
                  commands,
                  observers,
                  report_statistics=2)
```

In the following code snippet, we increase the infection rate by assigning a uniform distribution with much higher lower and upper bounds.

```
# Load the simulator from JSON file
simulator = parser.parse_simulator()
```

```
# Change the infection rate here (or just change in the respective JSON
    file and then parse the simulator)
from distributions import Uniform_Disease_Property_Distribution
simulator.disease_properties.infectious_rate_distribution = \
   Uniform_Disease_Property_Distribution(parameters_dict={"upper_bound":0.95,
       "lower_bound":0.9})

# Generate the simulation model
simulator.generate_model()

# Load simulator's data from JSON file
end_time, spread_period, initialized_infected_ids, _, observers =
    parser.parse_simulator_data()

# No policy is required
commands = []

# Execute the simulation
simulator.simulate(end_time,
                  spread_period,
                  initialized_infected_ids,
                  commands,
                  observers,
                  report_statistics=2)
```

The results of both lower and higher infection rates are shown in Figure 1b. A significant displacement in the peak of the curve is observable that exactly matches our expectations from changing the infection rate.

```
from utils import Health_Condition
data_2 =
    observers[0].get_disease_statistics_during_time(Health_Condition.IS_INFECTED)

from plot_utils import Plot
Plot.plot_multiple_lines(data_1[1], [data_1[0], data_2[0]])
```

6 Decrease immunity

In this section, the immunity is decreased, and the results of the simulation are shown in the following figure. With this amount of immunity, almost every person should get infected. The pandemic curve will also not become flat since there is a small generated immunity after catching the infectious disease for the first time. The results of this experiment are observable in fig. 1c.

```
# Import Parser
from json_handle import Parser
```

```
parser = Parser('test')

# Load the simulator from JSON file
simulator = parser.parse_simulator()

# Change the infection rate here (or just change in the respective JSON
    file and then parse the simulator)
from distributions import Uniform_Disease_Property_Distribution
simulator.disease_properties.immunity_distribution = \
   Uniform_Disease_Property_Distribution(parameters_dict={"upper_bound":0.03,
       "lower_bound":0.02})


# Generate the simulation model
simulator.generate_model()

# Load simulator's data from JSON file
end_time, spread_period, initialized_infected_ids, _, observers =
    parser.parse_simulator_data()

# No policy is required
commands = []

# Execute the simulation
simulator.simulate(end_time,
                  spread_period,
                  initialized_infected_ids,
                  commands,
                  observers,
                  report_statistics=2)

# Plot the results
from utils import Health_Condition
observers[0].plot_disease_statistics_during_time(Health_Condition.IS_INFECTED)
```

7 Quarantine all families

The simulator is capable of enforcing quarantines based on families, in addition to persons and communities. In this part, we impose a full quarantine over all the families and observe the results. This should have the same effect as quarantining all the people. The result of this quarantine is depicted in Figure 1c.

```
# Import Parser
from json_handle import Parser
parser = Parser('test')

# Load the simulator from JSON file
```

```python
simulator = parser.parse_simulator()
simulator.generate_model(is_parallel=False)

# Load simulator's data from JSON file
end_time, spread_period, initialized_infected_ids, _, observers =
    parser.parse_simulator_data()

# Build a policy
from datetime import timedelta
from commands import Quarantine_Multiple_Families
from conditions import Time_Point_Condition

commands = [Quarantine_Multiple_Families(
                  condition=Time_Point_Condition(Time(timedelta(days=15))),
                  ids=[i for i in range(len(simulator.families))])]

# Execute the simulation
simulator.simulate(end_time,
                  spread_period,
                  initialized_infected_ids,
                  commands,
                  observers,
                  report_statistics=2)
```

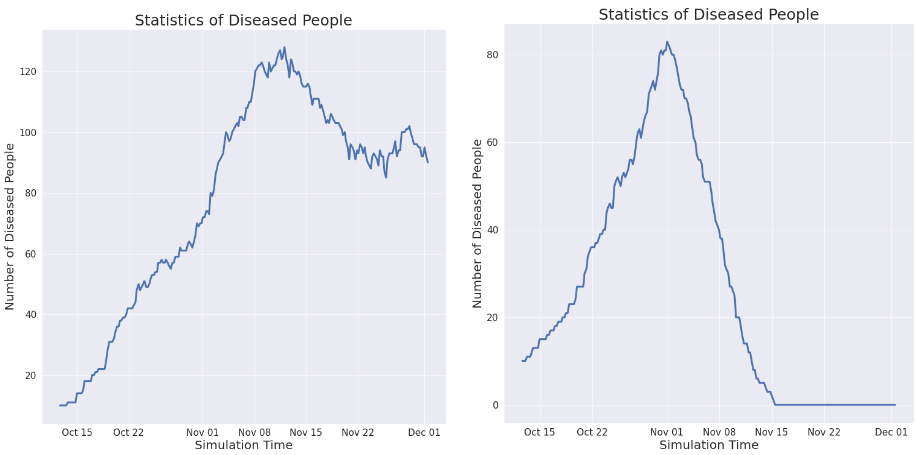

(a) A plain simulation run- without interference- (left) and enforcing quarantine on everyone (right).

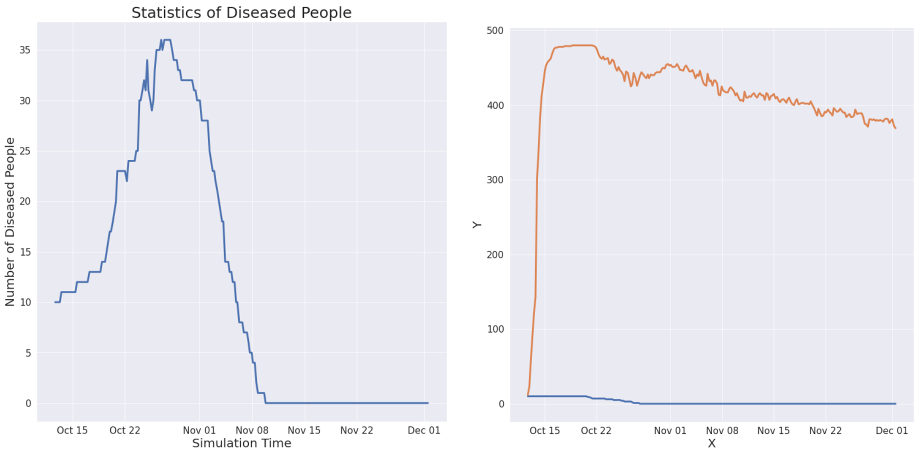

(b) Enforcing quarantine on infected people (left) and effect of the infection rate (right).

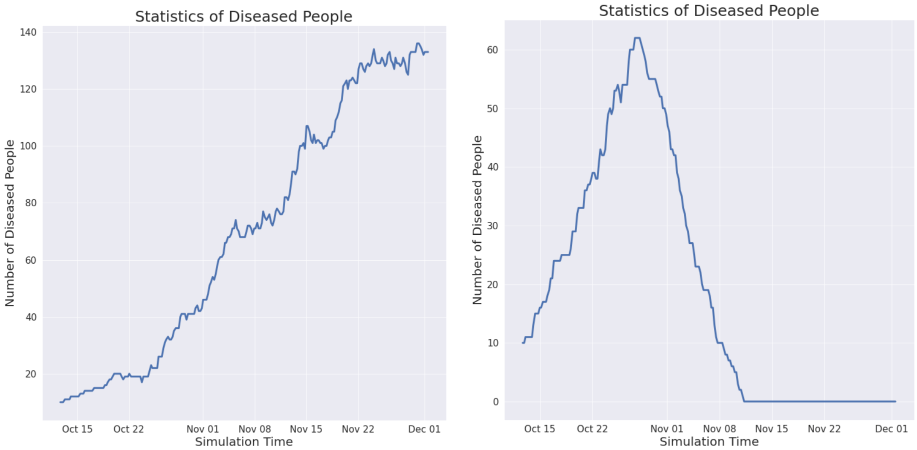

(c) Effect of reducing immunity (left) and enforcing quarantine on all families (right).

Figure 1: The results of observer plots
