# Supplementary material for: Pyfectious: An individual-level simulator to discover optimal containment policies for epidemic diseases
Source: PLoS Comput Biol. 2023 Jan 23;19(1):e1010799. doi: 10.1371/journal.pcbi.1010799 (PMC9894541; doi:10.1371/journal.pcbi.1010799)
Supplement: S4 File — Further experiments have been developed and presented in this file, in order to demonstrate the simulator’s ability to cope with a variety of given criteria. The experiments are fundamentally the same as the main text but vary in terms of parameters like population size or society’s structure. (PDF) [file pcbi.1010799.s004.pdf]

S4 Additional Experiments

In addition to the experiments presented in the main text, further experiments have been developed here in order to demonstrate the simulator's ability to cope with any given criteria. The upcoming experiments are fundamentally the same as the main text. However, the following differences in the configuration files are notable.

1. Incubation and disease period: the disease period and incubation rate are derived from uniform distributions of lower values (incubation period: Uniform [1, 2] and disease period: Uniform [5, 9]), as opposed to a normal distribution.

2. The population has a more simple structure but the same size, which means that people are divided into large communities compared to the main paper experiments.

3. The simulation duration is shorter than before, four months instead of ten months in the main experiments.

The results presented in Figure 1, Figure 2, and Figure 3, imply that the simulator is flexible with regard to various population and disease structures. Moreover, as appears in the mentioned experiments, our inference in a larger population is quite the same as what we observe in a simpler structure, indicating that the simulator is expandable to any population size if enough information is available about the overall structure.

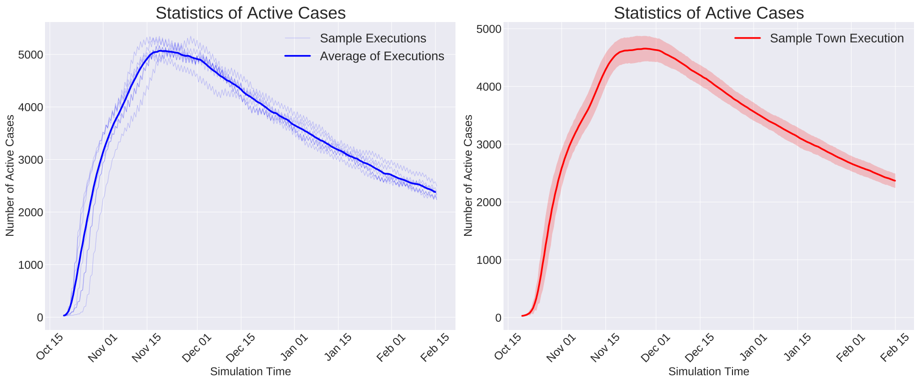

(a) Left) Normal executions of the sample city. Due to the stochastic characteristics of the simulation, each run is slightly different compared to the others. Therefore, the blue line is introduced as the average of all executions, smoothed by a moving average of windows size 2. Right) A plain execution of the simulation without any interference, i.e., the virus spreads from the initially infected people to others without the presence of any prevention measures. Since the simulation has a stochastic nature, the error bands are displayed as a confidence measure when making deterministic conclusions.

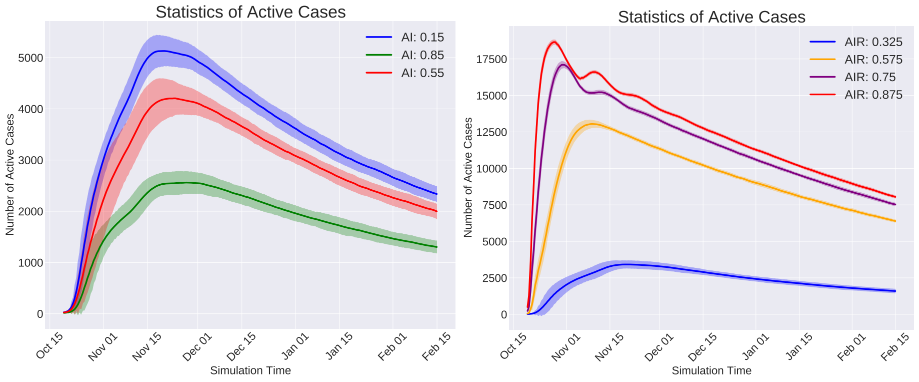

(b) Left) The effect of the immunity rate on the simulation. In this set of experiments, the immunity rate is sampled from three different uniform distributions. The results indicate that larger immunity rates give rise to flatter curves. Note that AI (Average Immunity) is the mean of the uniform distribution from which the immunity rate of each curve is sampled. Right) Effect of infection rate on the statistic of active cases. Each curve corresponds to a specific infection rate distribution. The results indicate that the larger the infection rate gets, the higher the spread slope will be. As a result, it takes less time for the number of active cases to reach its peak. Notice that AIR stands for the Average Infection Rate, which is the mean distribution from which the infection rate is sampled.

Figure 1: First part of the additional experiment results, including disease properties and normal executions.

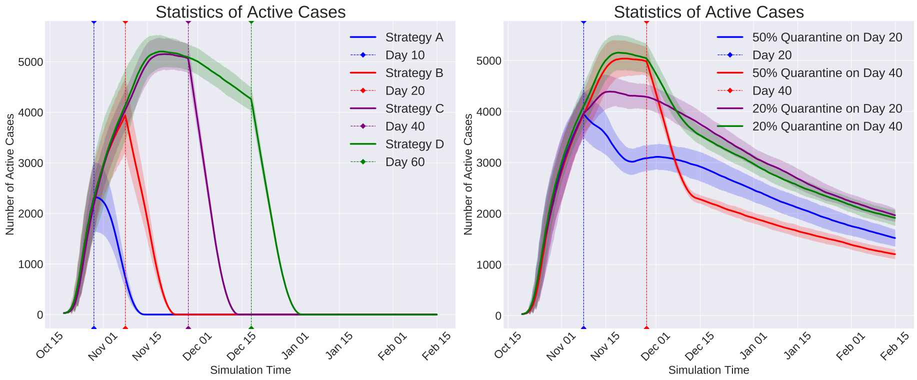

(a) Left) Quarantine the currently infected people activated by time point conditions. This experiment focuses on enforcing universal quarantines on a specific day of the simulation. Quarantines may be applied at any time point during a simulation. In this figure, the quarantines are mandated both before and after the active cases' curve reaches its maximum value. A, B, C, and D strategies enforce a quarantine after 40, 60, 100, and 200 days after the beginning of the outbreak. Right) Quarantine the currently infected people activated by time point conditions and detection error. This experiment focuses on enforcing a probabilistic quarantine after a specific number of days. In this experiment, the process of quarantining the infected people suffers from an error in the detection of active cases; therefore, changing the last part's deterministic nature into a probabilistic one.

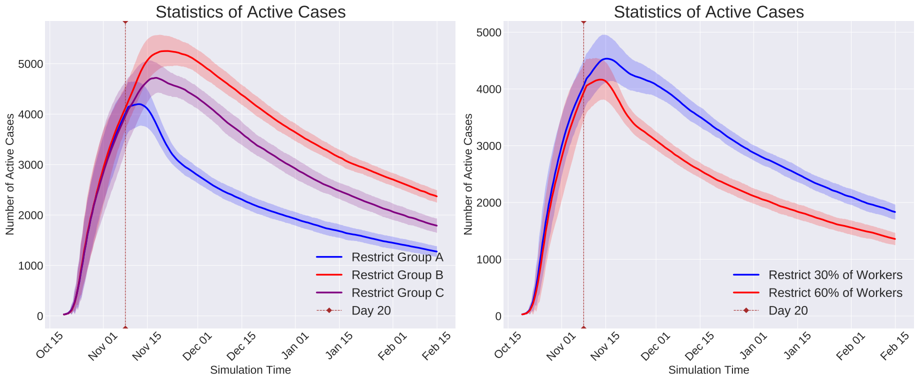

(b) Left) Quarantine specific sectors of society. Aiming at the importance of quarantining various economic sectors of society, this experiment is dedicated to displaying the outcomes of this type of quarantine on the population and the graph of currently infected cases. Group A includes all the workspaces of any size. Group B consists of gyms, restaurants, and cinemas, while group C is focused on more public groups like malls and public transportation. Right) Quarantine specific roles of the society. The simulator is also capable of contracting the virus spread by enforcing restrictions on certain roles in society. Specifically, in this experiment, we quarantine only workers of any kind, e.g., industry and offices. The effect is quite tremendous since the workplaces, in general, have a considerable transmission potential due to highly possible and repetitive close contacts.

Figure 2: Second part of the additional experiment results, including exemplary restriction measures.

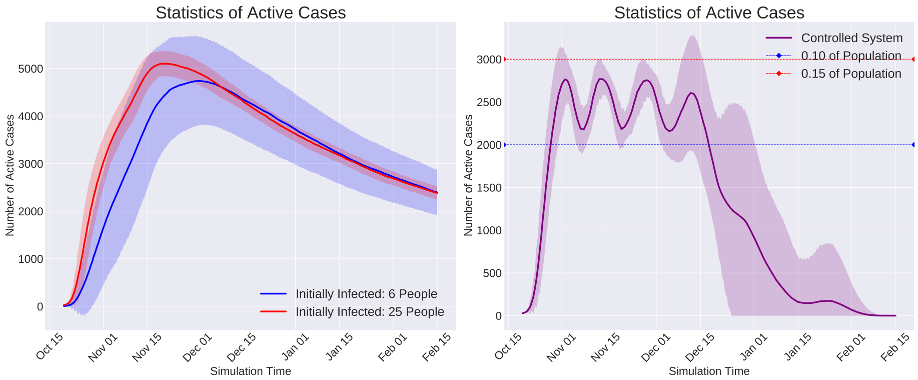

(a) Left) The effect of the number of initially infected people. The confidence intervals are expectedly wider for smaller initially infected set because it results in some communities without an initial spreader and consequently a less homogeneous spread of the disease. Right) The bang-bang controller. In this experiment, two different conditions are in place. One triggers quarantining the infected people; the other one triggers lifting the quarantine. Whenever the ratio of currently infected cases to the population reaches 15%, quarantines are enforced, and when the ratio declines below 10%, the quarantine is lifted.

Figure 3: Last part of the additional experiment results, including bang-bang controller and effect of initially infected individuals.

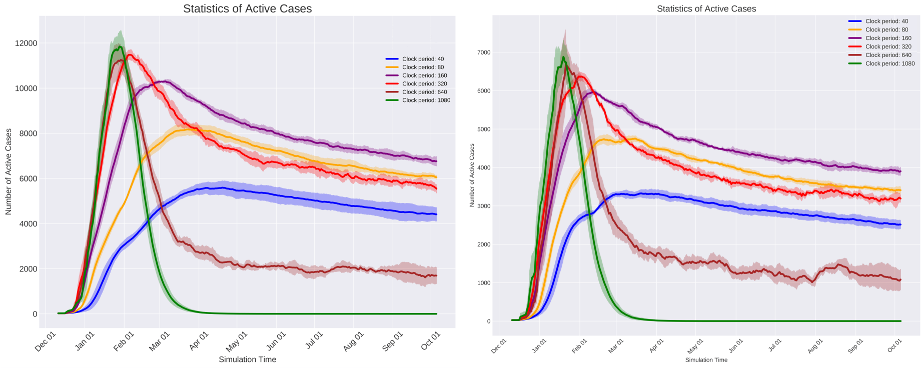

(a) (b)

Figure 4: Experiments demonstrated here are focused on simulator's unvarying performance w.r.t. system resolution (clock period), either when applying various control policies or by changing the spread period. The population structure and other details related to these experiments are the same as the main experiments, with a population size of 20k people. Part (a) represents several simulations only different in the value of spread period. Part (b) is the same experiment for 10k population size, suggesting that invariant behaviours w.r.t. system resolution hold for large enough populations. Here, this behaviour shows more uncertainty since the population size is smaller. Besides, this plot demonstrates that good policies generalize relatively well w.r.t. population size.
