# Supplementary material for: Pyfectious: An individual-level simulator to discover optimal containment policies for epidemic diseases
Source: PLoS Comput Biol. 2023 Jan 23;19(1):e1010799. doi: 10.1371/journal.pcbi.1010799 (PMC9894541; doi:10.1371/journal.pcbi.1010799)
Supplement: Attachment — Submitted filename: Pyfectious_response_to_reviewers.pdf [file pcbi.1010799.s005.pdf]

Authors' Response to Reviews of

Pyfectious: An individual-level simulator to discover optimal containment policies for epidemic diseases

A. Mehrjou, A. Soleymani, A. Abyaneh, S. Bhatt, B. Schölkopf, S. Bauer
Submitted to the journal of PLOS Computational Biology

RC: ***Reviewers' Comment***, AR: Authors' Response, ☐ Manuscript Text

We thank all reviewers for their valuable feedback, that helped us improve the manuscript significantly. We tried to answer all concerns of the reviewers and edited the manuscript and the codebase repository accordingly. We would like to point out a few general points here: (1) The numbering of equations, sections, and figures are based on the new (revised) manuscript unless otherwise stated. (2) All edited and added parts in the revised manuscript are colored red for better visibility.

1. Reviewer #1

AR: We would first like to thank the reviewer for the positive comments on the novelty of our work and the value of the presented approach. In addition, the reviewer's suggestions were very insightful and helped us improve the paper. Below we include our responses to the major comments.

RC: ***My overall impression of the paper was that it had an abundance of technical details about the software yet could have benefitted from a more focused narrative to support the authors' claims about the work.***

AR: This work introduces a new software package which is based on a basic principle that every involved object is a random variable and consequently the whole population (e.g. city) becomes a complex large random variable. We would like to emphasize that the probabilistic nature is not an added component to e.g. model the uncertainty of a certain parameter. It is a fundamental design principle that holds in every component (e.g. population generator, disease model, the time evolution of the disease propagation, etc) and every level (e.g. individuals, subcommunities, communities, etc.). In light of the received reviews, we have focused our narrative to better highlight the main ideas and benefits of our novel design.

RC: ***The authors made three broad claims: 1. Their approach to building a synthetic population (and other aspects of models) using a hierarchical random variable is more robust against sampling artifacts and gives confidence bounds for decisions based on the simulation results. 2. The simulator is used as the environment of a reinforcement learning problem to find the optimal policies to control the pandemic. The obtained experimental results indicate the simulator's adaptability and capacity in making sound predictions and a successful policy derivation example based on real-world data. As an exemplary application, our results show that the proposed policy discovery method can lead to control measures that produce significantly fewer infected individuals in the population and protect the health system against saturation. 3. Comprehensive documentation and code-snippets of the use cases are provided as Jupyter notebooks to facilitate a quick start in running experiments with an arbitrary setting for epidemic researchers or policymakers.***

AR: Thank you for your detailed comment on the claims of the paper and for bringing these references to our attention. In the following, we address these points connected to each claim.

RC: ***For claim 1, while I did appreciate the explicit descriptions of the probabilistic aspects of their approach, stochasticity in agent-based models (ABMs) is very common, and there is abundant literature for sampling approaches that explicitly aim to produce confidence bounds from ABMs, either for propagating input uncertainties with ABC approaches (e.g., Beaumont 2019), Bayesian optimization of stochastic simulators (e.g., Binois et al. 2018), iterated Bayesian filtering (e.g., Pei et al. 2018), and many others (e.g., Thiele et al. 2014). There is also the question about whether their approach improves on other methods for synthetic population construction, which also have stochastic aspects to them, e.g., Gallagher et al. 2018, Cajka et al. 2010, and more recently many others. In addition to not citing relevant comparators, the case was not clearly made that their contribution was novel and useful. In terms of uncertainties and how they can be utilized in supporting decision-making, I would point the authors to work in the robust decision-making (RDM) literature for examples (e.g., Lempert 2019).***

AR: Regarding the stochasticity in agent-based models (ABMs), we acknowledge the fact that some level of uncertainty has been embedded in some of these models. We also stated this explicitly in our original submission. The unique aspect of our work is in our *stochastic-first* approach where for each specific component (e.g. population, disease, etc) and every heterogeneous level (e.g. individual, communities, etc) uncertainty is captured. Furthermore, this stochasticity is probabilistic rather than a mix of deterministic and random factors (which dominate previous ABMs). Such comprehensive probabilistic modeling and simulation is rare to the best of our knowledge, and implementations tend to be bespoke rather than general. In combining a *stochastic-first* approach with a simple implementation in a widely-used programming language we believe our work to be unique and novel. Stated differently, the probabilistic-first approach makes the whole simulation environment (population and disease) a random variable and allows us to make a full probabilistic interpretation of the derived conclusion to be robust against variations in the parameters. This probabilistic-centered approach allows for rich extensions for both theory and practice.

Regarding the use of Approximate Bayesian Computation for posterior sampling (e.g. [1, 2]), we want to emphasize that we have not claimed a novel Bayesian inference method in this paper. However, in being probabilistic centered, our approach is readily extensible to Bayesian or frequentist inference - a feature not available in most ABMs. The main focus of this work is on the development of a simulator that is rich enough to be used as an RL environment. The parameters of the simulation such as the population setting or disease characteristics are assumed to be known by the experts or are estimated by standard Monte Carlo Bayesian inference techniques in the absence of a known likelihood. Similarly, even though our simulator has internal stochasticity, the input replicates as described in [3] does not occur in our model as the input to the simulator (population and disease properties) are also random variables. The sensitivity analysis of [2] could be relevant to the policy discovery stage of our work as the sensitivity of an outcome (e.g. number of deaths) to a certain parameter can be a proxy to the gradient of that outcome with respect to that parameter. However, we considered policy discovery a general search in an arbitrary space of policies. Even though sensitivity analysis can be useful to adjust a parameter around its current value, it is a local approach and cannot directly be used when the search space is discrete, for instance, switching from one policy class (e.g. quarantine) to another policy class (e.g. quick vaccination). Moreover, the locality of sensitivity analysis makes exploring even in the continuous search spaces (e.g. the continuous-valued parameter of some policy class) quite slow when a more favorable parameter lives far away from the current value of the parameter in the search space. Since selecting parameters is not a core objective of Pyfectious, we derive the required parameters for population and disease from real-world statistical data related to cities that have experienced the spread of the pandemic and have organized the data properly. For instance, in **General experimental setup** under our experiments section, you can see how the parameters for both the disease and the population are extracted from real-world sources.

Regarding the individual-level population generation, our approach is similar to [4] in the sense that we both

construct the population of individuals to support heterogeneity as opposed to mechanistic ODE/SDE -based compartment models whose applicability in biological models requires assumptions that may not hold in many cases as stated by [5]. However, the two works remain distinct in many angles some of which we enumerate in the following. The contact network in [4] is constructed based on the actual hospitalization records from Stockholm County, Sweden where the nodes are individual patients and the edges between two nodes mean two patients being in the same ward at the same time. Our population generation approach is far more generic and is actually part of the novel contribution of our work. Instead of manually constructing the population using real-world data, our method mimics the process by which a population is structured in a city. Our algorithm receives the number of individuals in a city and some generic information about the city structure such as the number of restaurants and schools which are generally called communities and the age pattern of the households. Then, through an inference process (see the **community assignment** section in the manuscript), the individuals are assigned to each community to form the structured population. Notice that assigning individuals to communities is not a trivial algorithm as individuals have relations with various communities and several constraints need to be satisfied at the same time. Hence, it can be viewed as solving a high-dimensional latent-variable model with heterogeneous probability distributions. Moreover, [4] considers only hospital wards as a location for disease transmission while Pyfectious's users can define arbitrary communities that correspond to real-world communities (e.g. schools, stores, restaurants, public transports, etc) in which contacts may occur. We would like to emphasize again that generality was one of the main design principles of Pyfectious, and as can be seen in the provided examples, defining communities such as schools, hospitals, restaurants, households are a few lines in a JSON file that contains the instructions for the simulation. Regarding the iterated filtering approach, we reiterate here that the focus of Pyfectious was not a new inference method, rather developing a rich RL environment that is probabilistic at all levels.

Pyfectious consists of three major components which are the population generator, the disease model, and the simulator. The first two components build the static parts that define the contact network among the individuals and different properties of the disease such as virulence etc. The simulator component carries out the dynamic part which takes the contact network of the population and along with the disease properties as input and evolves the propagation of the infection in the population. The references [6, 7] pointed out by the respected reviewer concerns the first component, i.e., the population generator of Pyfectious. We acknowledge the overlapping parts between our population generator algorithm and these methods especially [7] but would like to enumerate the directions in which our work differs in the following.

The occupation of individuals results in different types of interaction in the same communities. For example, even though both teachers and students share the same building location-wise, students have closer contact with each other than teachers. We call these communities that are formed inside the bigger communities, subcommunities. While [7] assigns individuals to locations based on their properties and occupations, the population generator of Pyfectious has the capability to model the subcommunities within each location as well. We found this feature important in the simulation of disease propagation as some of the occupations act as hubs inside the communities. For example, the security guard at the door of an institute has contact with every single person. If security guards get infected, they can thus potentially infect many individuals while a researcher who spends most of her working time in an isolated office can infect far fewer people. We elaborate on this capability of Pyfectious compared with other existing works at the **Generality** paragraph under the **Population model** in Section 1.

The other difference is the seamless integration of our population generator with the simulator. Every individual is assigned a daily schedule based on age, occupation, and other properties. As stated before, Pyfectious is probabilistic at every level which is also true for the schedules. Except for the activities whose starting and end times are fixed (e.g. attending school), the timing of other activities (e.g. going to a restaurant) are random variables with certain distributions (notice that all distributions used in Pyfectious can

be user-defined for full controllability). Such a detailed schedule assignment is absent in [6, 7]. We explained this point in the **Population model** item in Section 1.

The final point of difference we would like to emphasize is the programming language of Pyfectious, which is Python while [7] is implemented in R. We believe, given the absolutely dominant use of Python in the machine learning community, the pythonic implementation of Pyfectious makes it more convenient as an RL environment for further research.

In summary, many of the existing ABMs are specifically designed for a certain scenario or a particular dataset while Pyfectious is a general framework with simple and minimal design principles that can be adjusted to almost every scenario. Moreover, in existing works, the stochasticity often appears in a Bayesian posterior estimation of uncertain parameters which is a relatively small subset of all involved parameters of the model while the structure of the population is kept fixed. Our model is probabilistic centered where the whole population and disease models can be seen as hierarchical random variables which makes it easier to take into account uncertainty at every level not only restricted to the parameters. This has become feasible by numerous novelties in the population generation and time evolution phases that allowed Pyfectious to construct a consistent and realistic population without the need of receiving detailed information about the internal structure of the population.

RC: ***There was very little in the manuscript to support claim 2. Having said that, if the manuscript were to focus on using the software as part of a fully developed reinforcement learning application, applied to a real problem rather than a single proof of concept model that was provided, I think it could be compelling. The simple examples provided throughout Section 4 serve as verification of function rather than novel, or compelling use cases, and would likely be more appropriate for a software tutorial.***

We acknowledge your point that more experimental results on the use of Pyfectious as an RL environment would be more compelling. However, this work is mainly focused on the presentation of the ideas behind the design of Pyfectious that makes it a rich RL environment. In addition to detailed population generation, the monitoring and control components are designed with extensive flexibility, such that a very rich policy space is created. From this large policyc space, the agent can learn non-trivial course of actions to contain the spread of infection. For example, the agent can learn a trade-off between monitoring strategy (e.g. exhaustive daily testing) and strict control measures (e.g. complete lockdown.) An optimal strategy may be testing a certain group of people conditioned on their contact history and employing a targeted quarantine measure that keeps the infection under control, while the society keeps functioning. The policy space of the RL agent in Pyfectious is so large, that it will allow agents to explore trade-offs, whose consequence is hard to envision for humans. In Pyfectious, it is up to the user how much freedom one wants to give to the RL agent in exploring for possible policies. Hence, due to prior knowledge or limited computational resources, a human policy maker may choose a fixed policy and only let the RL agent fine-tune a few parameters around their nominal values. This is especially useful when the implementable policies in a certain society are limited and the policymakers need to find the right way to implement that policy (e.g. when to start the quarantine and how long to keep it). The possibility of such modifications is a core design principle of Pyfectious. We showcased the policy discovery for a limited policy class where a parameter of interest is fine-tuned using an uncertainty-driven learning method. Searching in a larger space of policies will probably need more sophisticated RL methods and certainly much more computational resources. We hope the richness and straightforward application of Pyfectious whose interface is consistent with the other RL environments, encourages the RL community to develop policy discovery algorithms tailored for epidemiological questions.

RC: ***R1C3: I agree that the authors did provide sufficient support to claim 3.***

AR: Thanks for recognizing the supporting evidence as being sufficient for this part.

RC: ***R1C4: I found the literature/model review utilizing Tables 1-4 in the introduction underdeveloped and not particularly helpful in contextualizing the Pyfectious contributions. I'd suggest more clearly describing the criteria used for choosing the cited examples and defining the assessments of each (e.g., how was detail level defined?).***

AR: We tried to include a diverse set of highly-respected and used simulators in terms of attitude and simulation methods in different categories of the literature, e.g., epidemiology [8, 9], artificial intelligence [10], social network [11], economics [12], etc. Some of them were retrieved by running queries on the google scholar database finding the recent highly-cited simulation studies. More discussion about the novelty of our simulator and comparison with existing works in connection with Tables 1 to 4 was added to Section 1 of the revised manuscript.

RC: ***The writing lacked focus and preciseness at times and should be improved for epidemiological content (e.g., see below) and overall clarity (e.g., pg. 4: "Many existing simulators have been developed for a particular population that is determined by their hyper-parameters.").***

AR: We agree with the reviewer and have modified the text in Section 2 to help improve the clarity and also have contextualized our work compared to previous studies.

RC: ***I've included some additional more minor points below.***
Minor points:
pg. 2: "Social distancing policies are often based on experts' common sense and previous experiences in partially similar conditions [1]. For instance, vaccination [3], travel restrictions [4], school closure [5], and wearing protective instruments such as masks [6] were used during SARS-CoV1 in 2003 whose effects were evaluated through several studies." –> Since vaccination is not a social distancing policy, I suggest removing it from this sentence about the implementation of social distancing policies.

AR: The sentence was modified in the revised manuscript. We appreciate the comment and, indeed, vaccination does not fit into the same category as other social distancing measures.

RC: ***pg. 7: "edges of a background timer" –> This concept ("edge" in the context of the timer) needs clarification.***

AR: A clarification footnote was added.

RC: ***pg. 7: "Infection Event" and "Incubation Event" –> For clarity, should these be renamed to something more indicative of the fact that the infection and incubation end when they occur?***

AR: We had noticed this point before the submission. Since the events are activated during infection and incubation time, but are triggered at the end of the respective periods, we thought naming them as something like "End Incubation Event" could lead to more confusion about the nature of the events. We admit that the provided information about the event may have been insufficient. Hence, we improved the descriptions in the revised manuscript to eliminate the potential confusion.

RC: ***pg. 8: "We divide the software system into three separate components that can be considered independently: 1) population generation, 2) disease propagation, and 3) time management." –> At the end of the Introduction section, there was an emphasis on the "two processes in the developed software package," the Population Model and the Propagation Model. It might be helpful to either relate the three components to the two processes or consistently refer to one of the organizing structures throughout.***

AR: Thanks for noticing this nuanced point. We changed the beginning of the section **Software architecture for Pyfectious** to relate these two. In summary, the population and propagation models are more abstract

divisions of involved processes. These processes are implemented as three components where the disease model and the time evolution component are needed to realize the propagation model in the population.

RC: ***pg. 12: Regarding the assignment of the profession, it seems that the order of profession assignment would matter but there was no discussion of how the professions are ordered. Also, the notation at the end of the "Special case of profession assignment" paragraph is unclear.***

AR: The professions are not ordered. The assignment of individuals to professions is probabilistic where the probability is calculated based on the fitness score of that individual for that profession. In other words, the order of likelihood of professions is different for each individual depending on her attributes. For example, individuals of younger ages are less likely to be assigned to professions like a teacher or university professors. The pointed-out paragraph explains our method to assign unique professions to individuals by making the fitness score of those individuals for other professions extremely negative once the individual is assigned a profession. We revised that paragraph to improve its clarity.

RC: ***pg. 12: All the edges appear to be directed in the connectivity graph so in describing the family connections I'd suggest using the same terminology from the Algorithm 2 pseudocode and indicating that the edges are bidirectional. Also, how are the α_{ij} and β_{ij} meant to be chosen for the Beta distribution, beyond just that the "probability mass is concentrated around 1 for $i = j$ and around smaller values for $i \neq j$"?***

Inter-subcommunity edges are indeed directional but are only depicted as bidirectional in Figure 3 for more convenient illustration. The essence of modeling interactions with directional edges is explained in Figure 2.

All hyper-parameters are learned by Bayesian inference methods (e.g. Monte Carlo-based sampling approaches) that also allow incorporating expert priors. More information about setting disease and other hyper-parameters can be found in Section 4.1 of the paper.

RC: ***pg. 14: What are the units for infection rate? Or is it a probability, given it is constrained to be between 0 and 1? I'd suggest clarifying the name and the definition.***

AR: The infection rate is normalized to be between 0 and 1. It is unitless but can be interpreted as a binomial probability that tells how likely the transmission is once two individuals are in contact. It can also take a frequentist interpretation. For example, $r = 0.4$ means that, out of every 10 contacts, roughly 4 of them lead to a successful transmission of infection. We elaborated more on the interpretation of infection rate in the section **Disease transmission between two individuals** of the revised manuscript.

RC: ***pg. 14: Is the transmission potential (γ) modeled as a Gamma or Beta distribution? The text appears to be inconsistent.***

AR: Thanks for catching this typo. Similar to the infection rate, this parameter is also a random variable with a Beta distribution. We corrected the typo in the revised manuscript where the transmission potential is described in section **Disease transmission between two individuals** of the revised manuscript.

RC: ***pg. 15: "We propose a novel mixture event/clock-driven method to bring together the benefits of both worlds." –> This is not necessarily novel, e.g., see North et al. 2013.***

AR: Thanks for bringing this work to our attention! A sentence, characterizing the novelty of our approach compared to [13] is added to the paragraph.

RC: ***pg. 15: "The queue of events is formed similar to the event-based methods, but the pulses of a background clock determine which events are effective in the outcome." –> This sentence is unclear as to what "which events are effective in the outcome" means.***

AR: A footnote for further illustration is added to this sentence.

RC: ***pg. 15: "Transition event Each transition event is associated with a certain individual, and it is triggered when that individual changes its location from one subcommunity to another. The change of location is implemented by changing the weights of the edges connected to an individual in the connectivity graph." –> Two elements that I think need clarification. 1) Are locations and subcommunities to be thought of as interchangeable concepts? 2) How do edge weights change?***

AR: Locations are considered for community recruitment, whereas subcommunities are involved in the evolution of the simulation. The reason is that the location of the households to which an individual belongs affects the communities that she can be assigned to. The change of location is implemented by changing the weights of the edges connected to an individual in the connectivity graph of the corresponding community from zero to γ_{edge} and vice versa.

RC: ***pg. 22: "measuring the required time for the model generation and simulation phases" –> Was the concept of "model generation" introduced previously?***

AR: Model generation refers to the generation of the population that creates the underlying graph for disease transmission. As a new term is not needed here, we replaced "model generation" with "population generation" in the revised manuscript.

2. Reviewer #2

RC: ***The authors define a principled, extensive, open-source environment for running epidemiological simulations, with the ability to define different population structures, conditionals, and event-driven milestones. However, it's not clear from the repository structure how one would go about reproducing the results found in the paper. From the Plos guidance: "A prerequisite for publication is that the results described in the paper must be reproducible when peer reviewers or editors choose to run the software on the deposited dataset using the parameters provided."***

We sincerely appreciate the feedback. To address the reproducibility issues, we added a reproducibility statement to the paper in Section 6 that provides a step-by-step manual for results reproduction.

RC: ***Major revisions:***
At present, the mapping between the results in the paper to a set of simulation configurations is not clear. Making reproducibility a challenge. e.g. which result does cluster_experiment_0 represent? Or inversely, which combination of configuration files represents the experiments highlighted in Figure 13? Provide such a mapping.

AR: Previously, we had provided files called `experiments.description` for each of the experiment sets. Apparently, the description files were not clear enough or lacked the expected clarity. To this end, we replaced each of the files with markdowns as a part of the git repository[1]. Furthermore, for the sake of reproducibility, we clarify the mapping between the experiments and the plots in the paper in markdown files placed under the experiment setups in `data/json` directory.

RC: ***A reconciliation needs to be made between the online documentation and intro guide via python notebook / Google Colaboratory and the supplemental getting started guide in the paper. The link on page 34 in the supplemental section is broken. This is of particular concern as it claims to provide code snippets from***

[1] https://github.com/amehrjou/Pyfectious/blob/develop/data/json/README.md

the paper.

AR: In order to make an accessible and hands-on tutorial, we moved our tutorials and examples from the `example` folder (the link previously mentioned on page 34) to our comprehensive Google Collaboratory tutorials are mentioned in our repository's README file. Utilizing Google Colaboratory, we aimed to offer our supplementary materials and code instructions in a collaborative environment without any particular hardware or software requirements. We hope this setup is more efficient and user-friendly compared to going through the files we already had in our GitHub repository.

RC: ***Given the substantial compute resources necessary to test the results, a reduction in barrier to entry could be made by providing cluster-specific launch scripts written for SLURM or other Distributed Resource Management Application API (DRMAA) compliant HPC management systems.***

AR: We have provided comprehensive README files to map experiments with the results presented in the paper. A link to our Google Colaboratory environment can be found in the Appendix along with a reproducibility statement that could help launch the files without a computational cluster. We hope these actions sufficiently address your concern regarding the entry point barrier. Moreover, the scripts we use to run the experiments are available in the *cluster* folder but need to be slightly adjusted for other clusters or for use on a personal computer, as described in the README file placed inside the folder.

RC: ***Overhead aside, the software does seem like a reasonably well engineered, modular and accessible entry point for those scientists interested in pursuing a counter factual based epidemiological question. It also represents a substantial amount of work. The paper is well written and describes the software well.***

AR: We thank the reviewer for this positive feedback and are glad that the principled design of our approach will be useful to infectious disease epidemiologists.

RC: ***Minor suggestions: One limitation appears to be the ability to explicitly track multiple strains of a disease and define a partial immunological relationship between the recovered states of such. Also the out-of-the-box software lacks the ability to define immune response waning as a function of time. Additionally modeling an asymptomatic or pre-symptomatic state would be an interesting modification as it relates to the stated observer functions. These would go along way in terms of addressing current epidemic related concerns.***

AR: We answer each of these concerns separately here. It does worth mentioning that while these concerns are referred to as minor, the modifications required to extensively address some of the requested features are beyond the scope of the current paper.

1. Tracking multiple strains of the disease: while we keep a record of the simulated disease using a `disease_manipulator` object, with little modifications, it is possible to keep multiple disease properties objects. Each of the objects would have the same overall construct, but parameters like immunity and infection rate are allowed to vary. However, one must define an initial infection rate for each of the strains, i.e., what percentage of the initially infected people have this specific variation.

2. Immune response as a function of time: A special and prominent capability of this simulator is the customization and extendability of all the attributes like immunity. Immunity can be given as a function of each individual's attributes given their property and environment situation (e.g., in the mentioned case, as a function of time, or in some other cases, different kinds of vaccinations create different levels of immunity.).

3. Asymptomatic and pre-symptomatic states: This feature is not directly integrated into the simulator's

core, but it has been imitated with quarantine policies, as presented in our experiments. Through imperfect quarantines, we pass an asymptomatic probability to address the infected people that may not be detected because they do not show any symptoms. For pre-symptomatic transmission, we have assigned a probabilistic transmission window that does not necessarily start with the symptoms. Changing this distribution makes one able to simulate pre-symptomatic transmissions.

3. Reviewer #3

RC: ***I think Pyfectious represents an interesting addition to the family of agent-based models for infectious disease policy evaluation. There are many that exist already however and this paper does not make a strong case for the novelty of the platform such that I think it merits publication in this journal in its current form.***

AR: We are happy to elaborate more on the novelties of Pyfectious that we also tried to emphasize in the revised manuscript. Between two major classes of epidemiological simulators, which are compartment and agent-based models (ABMs), Pyfectious belongs to the ABMs where instead of compartment-level ODEs/SDEs, the behavior of every individual is taken into account. One important aspect of Pyfectious is its holistic approach to stochastic modeling. We acknowledge the fact that some level of uncertainty has been embedded in some of the existing ABMs which we also stated explicitly in the original submission. The unique aspect of our work though is in our *stochastic-first* approach where for each specific component (e.g. population, disease, etc) and every heterogeneous level (e.g. individual, communities, etc) uncertainty is captured. Furthermore, this stochasticity is probabilistic rather than a mix of deterministic and random factors (which dominate previous AMBs). Such comprehensive probabilistic modeling and simulation is rare to the best of our knowledge, and implementations tend to be bespoke rather than general. In combining a *stochastic-first* approach with a simple implementation in a widely-used programming language we believe our work to be unique and novel. Stated differently, the probabilistic-first approach makes the whole simulation environment (population and disease) a random variable and allows us to make a full probabilistic interpretation of the derived conclusion to be robust against variations in the parameters. Moreover, the employed probabilistic-centered approach allows for rich extensions for both theory and practice.

Many of the existing epidemiological simulators propose inference approaches to estimate the unknown parameters of the model (e.g. see [1, 2]). It is worth mentioning that the main focus of this work is on the development of the simulator that is rich enough to be used as an RL environment instead of a new probabilistic inference approach. The parameters of the simulation such as the population setting or disease characteristics are assumed to be known by the experts or are estimated by standard Monte Carlo Bayesian inference techniques in the absence of a known likelihood. Since selecting parameters is not a core objective of Pyfectious, we derive the required parameters for population and disease from real-world statistical data related to cities that have experienced the spread of the pandemic and have organized the data properly. For instance, in **General experimental setup** under our experiments section, it can be seen how the parameters for both the disease and the population are extracted from real-world sources.

Another direction of work that can be related to Pyfectious especially in the policy discovery phase, is sensitivity analysis, e.g. in [2] where the sensitivity of an outcome (e.g. number of deaths) to a certain parameter can be a proxy to the gradient of that outcome with respect to that parameter. However, we considered policy discovery a general search in an arbitrary space of policies. Even though sensitivity analysis can be useful to adjust a parameter around its current value, it is a local approach and cannot directly be used when the search space is discrete, for instance, switching from one policy class (e.g. quarantine) to another

policy class (e.g. quick vaccination). Moreover, the locality of sensitivity analysis makes exploring even in the continuous search spaces (e.g. the continuous-valued parameter of some policy class) quite slow when a more favorable parameter lives far away from the current value of the parameter in the search space.

Focusing on the population synthesizer, Pyfectious stands out of the existing methods in various aspects. One design principle of Pyfectious has been its generality which makes it easily adjustable to every arbitrary population with any size or structure while many of the existing methods have been developed for a certain setting or dataset. For example, [4] constructs the population of individuals to support heterogeneity as opposed to mechanistic ODE/SDE -based compartment models. However, the contact network in [4] is constructed based on the actual hospitalization records from Stockholm County, Sweden where the nodes are individual patients and the edges between two nodes mean two patients being in the same ward at the same time. Our population generation approach is far more generic and is actually part of the novel contribution of our work. Instead of manually constructing the population using real-world data, our method mimics the process by which a population is structured in a city. Our algorithm receives the number of individuals in a city and some general information about the city structure such as the number of restaurants and schools which are generally called communities and the age pattern of the households. Then, through an inference process (see the **community assignment** section in the manuscript), the individuals are assigned to each community to form the structured population. Notice that assigning individuals to communities is not a trivial algorithm as individuals have relations with various communities and several constraints need to be satisfied at the same time. Hence, it can be viewed as solving a high-dimensional latent-variable model with heterogeneous probability distributions. Moreover, [4] considers only hospital wards as a location for disease transmission while Pyfectious's users can define arbitrary communities that correspond to real-world communities (e.g. schools, stores, restaurants, public transports, etc) in which contacts may occur. We would like to emphasize that generality was one of the main design principles of Pyfectious. As can be seen in the provided examples, defining communities such as schools, hospitals, restaurants, and households are a few lines in a JSON file that contains the instructions for the simulation.

Another novelty of Pyfectious is that the occupation of individuals results in different types of interaction in the same communities. In most of the existing simulators such as [7], the individuals who are assigned to a certain location, are homogeneously interacting with their community. All members of the school community (teachers, students, managers, etc) are at the same risk of infection because they share the same location. However, this is not a realistic assumption, in particular, for the infections that are transmitted with close contacts. To compensate for this, Pyfectious allows for defining different groups within each community (location) with different patterns of interaction. For example, even though both teachers of a school are in the same building, students have closer contact with each other than teachers. We call these communities that are formed inside the bigger communities, "subcommunities". While [7] assigns individuals to locations based on their properties and occupations, the population generator of Pyfectious has the capability to model the subcommunities within each location as well. In addition to different contact patterns, this feature is also important in the simulation of disease propagation as some of the occupations act as hubs inside the communities. For example, the security guard at the door of an institute has contact with every single person, if get infected, can infect many individuals while a researcher who spends most of her working time in an isolated office can infect far fewer people. We elaborate on this capability of Pyfectious compared with other existing works at the **Generality** paragraph under the **Population model** in Section 1 of the revised manuscript.

Another novelty of Pyfectious is the seamless integration of our population generator with the simulator. Every individual is assigned a daily schedule based on age, occupation, and other properties. As stated before, Pyfectious is probabilistic at every level which is also true for the schedules. Except for the activities whose starting and end times are fixed (e.g. attending school), the timing of other activities (e.g. going to a

restaurant) are random variables with certain distributions (notice that all distributions used in Pyfectious can be user-defined for full controllability). Such a detailed schedule assignment is absent in the highly used existing methods such as [6, 7]. We explained this point in the **Population model** item in Section 1 of the revised manuscript.

The final point of difference we would like to emphasize is the programming language of Pyfectious. Even though there are many recently developed python-based simulators, we notice many of the popular ones, especially those agent-based models with a comparable level of details to Pyfectious are implemented in other programming languages such as R [7]. We believe, given the dominant use of Python in the machine learning community, the Pythonic implementation of Pyfectious makes it more convenient as an RL environment for further research.

In summary, many of the existing ABMs are specifically designed for a certain scenario or a particular dataset while Pyfectious is a general framework with simple and minimal design principles that can be adjusted to almost every scenario. Moreover, in existing works, the stochasticity often appears in a Bayesian posterior estimation of uncertain parameters which is a relatively small subset of all involved parameters of the model while the structure of the population is kept fixed. Our model is probabilistic centered where the whole population and disease models can be seen as hierarchical random variables which makes it easier to take into account uncertainty at every level not only restricted to the parameters. This has become feasible by numerous novelties in the population generation and time evolution phases that allowed Pyfectious to construct a consistent and realistic population without the need of receiving detailed information about the internal structure of the population.

RC: ***The EpiSIMS platform (LANL) which you cite and GLEAM from Northeastern Univ and FRED from the University of Pittsburgh as well as EpiFast and EpiSimdemics from Virginia Tech all have similar manners of generating synthetic populations and much more elaborate and thus useful representations for disease progression and interventions. Many of these tools have been published on and in widespread use for decades.***

AR: Thank you for discussing these works, some of which we have already cited in our original submission. With respect to population generation and simulation, our unique contribution compared to these simulators is customization and extendability. We provide a simple-to-use toolkit for developing customized arbitrary characteristics of the population and disease propagation. As a compelling example, one can come up with the idea of considering public holidays and assessing their effect on the spread of the virus. Likewise, another one may define specific biweekly gatherings in some neighborhoods. To recap, the level of the details is definable and customizable by the user. Many other simulators tend to be far more restrictive and less customizable. The same scenario is true for the interventions and disease progress in a much more detailed way. Pyfectious has a general and extendable way to define `Conditions` and `Commands` which provides almost limitless opportunities to define new population monitoring and control measures. The interventions (policy enforcement) in the mentioned simulators are confined with the predefined integrated space of possibilities in the simulator, whereas in our case, arbitrary probabilistic even conditional policies, can be defined. For instance, EpiSimS intervention space lies in the classical scope of control measures consisting of quarantine/treatment/closures/social distancing/contact tracing; In addition to these primary policies, Pyfectious is capable of simulating arbitrary interventions such as applying contact tracing to individuals having ages between 10-15, who have already been contacted with at least two suspicious cases in a community whose household has more than five members.

Lastly, we would like to mention that many developments in packages such as EpiSIMS, EpiFast, and EpiSimdemics have been occurred gradually and throughout their uses by the epidemiology community. This

has resulted in an organic growth that does not have code design principles from computer science. Such extension to new and divergent use cases can be challenging. We hope Pyfectious v1.0 be a solid starting point with distinctive design principles from the existing simulators. The core principle which has affected the design decisions at every level of the development of Pyfectiousis its ultimate use as a rich and easy-to-use simulator where the richness is not traded for simplicity. The wholly probabilistic nature of the components of Pyfectious (population generator, disease model, daily schedules, etc) contributes to this principle so that the environment (both state space and action space) are rich enough that a non-human policymaker can find non-trivial policies in the vast space of possibilities that is extremely hard for a human agent to explore efficiently.

We hope these design choices help the adoption of Pyfectious not only in the epidemiology community but also in the community of machine learning (ML) and reinforcement learning (RL) researchers. We are committed to observing the issues and feedback from both communities to improve Pyfectious and maintain the software package that facilitates the ML/RL research in the space of epidemiology where the control measures are currently mostly proposed by human agents out of experience or known models.

RC: ***The breakthrough your tool represents is its efficiencies allowing for "simulation in the loop" style reinforcement learning as well as the wide array and variety of control measures. While this has also been done by researchers with the above-mentioned tools, increasing the efficiency of this process is important and may be significant. Focusing more on this aspect of the tool and less on the features would be useful for this manuscript.***

AR: Our contribution in this regard to the community is centered around the clock/event-based/observer idea. Whereas classical simulators are either clock-based or event-based, our method incorporates both. This idea allows us to have as much detail as event-based models have. Moreover, by enabling a settable clock period (i.e. the period in which one virus spread event occurs) have a multi-resolution system. On the theoretical ground, event-based models are the fastest models w.r.t. the evolution of time without losing any details that clock-based models usually suffer from.Pyfectious preserves this efficiency and also allows choosing the precision of the system w.r.t. the computational capacity of the user's computer by integrating a settable clock. Theoretically, in an irreducible environment, our method remains invariant to the system's resolution due to the ergodicity of the involved dynamics. However, irreducibility is not satisfied either in simulations or real-world scenarios; e.g., a person surviving a disease becomes immune to the disease. But asymptotic behaviors of ergodicity are observed in the experiments and are helpful for computational efficiency by preserving asymptotic behaviors of the simulation while having lower resolution (and therefore be more efficient). To illustrate this effect, we showed that the same qualitative conclusions can be made out of the simulations with a wide range of clock periods. In other words, a user with a restricted computational resource can make the same conclusion about the efficacy of a control policy as the user who has access to a much larger computational resource. A thorough discussion on this is added to the paper in Section 4.2.1.

RC: ***The features of this simulator are extensive, however, the main shortfall is that the disease model is very limited (seems to only represent infection and death, whereas hospitalization and symptomatic illness are equally if not more important). Additionally, the ability to specify disease parameters for different segments of the population is an essential part of COVID, and does not seem to be a part of this tool (e.g., mortality rates are much much higher for older individuals, who have different communities, etc.). Interventions beyond social distancing are important elements of outbreak control, and the relative timing of diagnoses and treatment are essential. None of the features appear to be in this tool, which I think will limit the use cases for this software and its overall adoption.***

AR: We took the following actions, divided into four major categories, to address your concerns related to disease

properties.

1. Presenting a more sophisticated disease model: To mitigate the limitations mentioned about our disease model, we added the following items: I) A hospitalization rate distribution, based on which infected people are sent to a hospital community where they stay until the final stage of disease, death or recovery, occurs. II) A hospitalization period, during which people are kept in the hospital and are released afterward; III) A pre-symptomatic period distribution, determining the period in which people are contagious but show no symptoms.

2. Different disease parameters for different segments of the population: We would like to emphasize that Pyfectious accommodates a much richer disease model than community-dependent disease parameters. Typically, the simulator only considers a fixed set of disease specifications for different segments of the population. Although it is important to make disease parameters specific to communities, we take a rather different approach by building more complex distributions in our disease manipulator class. For instance, based on the simulation expectations, one could come up with a distribution for mortality rate that takes into account both age and health condition of the involved people. In this way, we can introduce potentially unlimited ways of assigning disease parameters, not necessarily based on communities, but based on individual specifications. To justify this point of view, please consider a large nursing home facility. Within a community of this type, there are vulnerable elderly people that are more susceptible to infection; however, there are other groups of people like nurses and social workers that are not as vulnerable to infection- less mortality rate perhaps- but will fall into the same category of disease parameters if we assign certain parameters to communities. This example shows that the individual-dependent disease model is more expressive than the community-dependent disease model. The same example could potentially be extended to other roles/communities within the simulator.

3. The asymptomatic transmission state: This issue was also raised by Reviewer #2. A shortcut to cope with this concern is to imitate asymptomatic patients by overlapping the contagiousness period and disease period and bringing the contagious period ahead of the disease period , i.e., an infected person may be contagious but not detected until the symptoms appear. However, to further integrate this feature into our simulator, we added an asymptomatic state to the infected person's states, during which the person is capable of transmitting the disease but cannot be detected due to lack of symptoms.

4. Defining a hospital community: To address this issue, we defined an additional community called hospitals, where every infected individual is sent based on a Bernoulli probability distribution. This probability may also be referred to as the hospitalization rate. In the current version of the simulator, we assume that people who go to hospitals, cease every contact with the outside world, and therefore are not capable of spreading the infection further. Moreover, the fatality rate is defined to be relatively higher among this group compared to normal infected individuals as often more severe infected cases end up in hospitals seeking medical care.

RC: ***Assessing simulation performance in the rather narrow confines of 5K-30K size of the population is not enough to determine if the simulation performance is linear, at least 3 orders of magnitude are needed to assess scaling. In my experience, detailed agent-based simulations as listed above can simulate towns of this size considerably faster than 100s of minutes for 48 hours, but perhaps I have misunderstood the metrics. I think focusing a little more on the performance would benefit the paper. For example: How do the different "controllers" affect the run time? do certain commands impact the runtime more? What is***

the full run time for finding an optimal policy? Some of this is addressed in the final section of the paper, but seems more important than the limited time provided.

AR: A detailed discussion on this topic was added to the revised manuscript in Section 4.2.1. As mentioned in the added discussion, the provided plot is for 60 mins as the clock period, and gaining $T/60$ speedups is possible by having T as the clock period. Hence, 100s of minutes can be reduced to 10s of minutes yet enjoy consistent meaningful results. Figure 8a is only to prove the empirical partial linear relationship of the simulation time w.r.t. the population size. Furthermore, as requested, a practical demonstration of the computational overhead of control measures is added in Table 7. Plus, runtime for policy discovery is also added to Table 8.

When applying commands directly, or using them to build control policies, the runtime is hardly affected. This is because of the fact that all commands, conditions, and observer policies either use a single loop over the population or SQL queries to retrieve the required information. This insignificance is evident in Table 7.

RC: ***Several set of experiments are described in the paper, but only one "optimal" policy finding case study is presented. I'd be very interested to know what conditions in the population or disease might shift the found optimal solution. An exploration of these kinds of findings (both intuitive and counter-intuitive) would better showcase Pyfectious's benefits.***

AR: We appreciate your point and admit that the policy search aspect is interesting to illustrate. However, in this paper, we mainly aimed to introduce the software and its potential to the community rather than doing an in-depth investigation of the discovered optimal policies as a function of disease or population parameters. We hope Pyfectious provides the opportunity to study such questions in both epidemiology and RL communities with minimal overhead time and effort.

RC: ***To summarize, evaluating the performance in finding different optimal social distancing combinations in more detail would be useful to describe Pyfectious's strengths more. Some consideration of pharmaceutical control measures and the difficult tasks of differential timing of case identification and treatment is also important as it is VERY relevant to disease control***

AR: We appreciate your suggestions and believe these will be excellent points to study further. This paper is mainly intended to be an introduction of Pyfectious, the design ideas behind it, and its potential to be a reinforcement learning environment. The experiments were designed to showcase these points rather than being a comprehensive study to answer a particular research question.

4. Reviewer #4

RC: ***The paper describes Pyfectious, a Python package that allows simulation of a disease outbreak at the individual level. It allows creating a probabilistic model of population, simulation of a wide range of policies, and finding optimal control policies using a reinforcement learning framework. Developing an individual-level simulator (e.g., an agent-based model) for epidemiology could take a significant amount of time. The Python package described in this paper could be quite useful to public health researchers and allow them to focus their efforts in supplying realistic population parameters (demographics, communities, and connectivity) and evaluating intervention strategies instead of developing code and reducing time. The paper provides a reasonably detailed description of the simulation design and examples.***

AR: Thanks for pointing out the merits of Pyfectious! We hope this software to be useful for health researchers to quickly see the outcome of different control strategies and also for ML/RL researchers to design algorithms

that learn optimal strategies that are not easy for humans to find.

RC: ***I have the following questions:***
1. Page 14, the last line of "Transmission potential" mentions Gamma distribution and then uses Beta distribution. Could you please clarify?

AR: Thanks for catching this typo. Similar to the infection rate, this parameter is also a random variable with a Beta distribution. We corrected the typo in the revised manuscript where the transmission potential is described in Section 3.2.1 of the revised manuscript.

RC: ***Page 15, Transition event represents a change of location which is implemented by changing weights of edges connected to an individual in the connectivity graph. How are weights determined? Is it based on activity duration (i.e., the amount of time two individuals are at the same location at the same time)?***

AR: The change of location is implemented by changing the weights of the edges connected to an individual in the connectivity graph of the corresponding community from zero to γ_{edge} and vice versa. Plus, activity durations are captured by virus spread events (simulation clock).

RC: ***3. I was wondering if this package could be used to implement contact tracing, e.g.,*** 50% ***of the contacts of infected individuals could be traced and put in quarantine for 2 weeks?***

AR: Thanks for bringing up this idea that would be a valuable addition to the next versions of Pyfectious. Though not directly integrated into the software, Pyfectious is potentially capable of tracing contacts with slight modifications. That is, at the disease propagation stage, one can keep a record of people who have been in contact with diseased individuals. The methods for this purpose are already available and due to the modularity of Pyfectious, extending the core software to implement on-demand features like this is straightforward. We believe such extendibility is one of the core strengths of Pyfectious.

RC: ***4. Page 29, last paragraph, should value of 0.2 for column customers in the last row of table 7 mean that*** 20% ***of the customers are restricted and*** 80% ***allowed (instead of 20% allowed as mentioned in the text on Page 29)?***

AR: This was merely an error in describing the balance of restrictions (despite the correct description for the workers and students in the preceding line). Thank you very much for noticing it.

RC: ***5. What are the last two values (True and 1) represent when creating Community_Type_Role 'student_type' on Page 36, section 6.2.2?***

AR: The detailed description of these values and all other parameters can be found in the documentation of the code. For instance, to find the answer to this question, please see *population_generator.py* file[2]:

- is_profession (bool): Indicates if this role is a career.
- presence_prob (float): Probability of being present in this community, initiating with one, it changes whenever the workspace is subject to governmental restrictions or other environmental conditions.

RC: ***Here are some suggestions that could help improve the paper:***
1. Second line of the introduction states "While the first category involves medications and vaccination, the second approach, which is the main interest of the current work, concerns interventions on human communities to slow down the spread of the disease." I don't think, this differentiated pharmaceutical and

[2]https://github.com/amehrjou/Pyfectious/blob/master/src/population_generator.py#L591

non-pharmaceutical interventions clearly. Both approaches concern interventions on human communities to slow down the spread of a disease. As you mentioned in the first approach involves medications or vaccines and second approach involves reducing potential for disease transmission by behavioral interventions such as wearing masks, social distancing, or quarantining infected people.

AR: Thanks for your suggestion! We would like to emphasize that, by "pharmaceutical" we refer to the efforts in discovering medicine and vaccines. On the other hand, "non-pharmaceutical" refers to every other effort which intends to slow down the spread of the disease such as quarantining the infected individuals. In this categorization, the development of vaccines falls within pharmaceutical efforts while vaccinating the population is a non-pharmaceutical action. Please notice that, for vaccines, we made a distinction between the development phase (pharmaceutical) and the application phase (non-pharmaceutical). The former includes efforts from the biological and chemical R&D phase to clinical trials. The latter assumes the vaccine is developed and has a certain effect in reducing the probability of getting infected in every contact. Hence, studying the effect of vaccines amounts to investigating the outcome of a situation where there is a shortage of vaccine supply or the population is not fully vaccinated. From this perspective, while developing the vaccine is a pharmaceutical effort, getting vaccinated is a behavioral intervention and falls within the non-pharmaceutical category. We made this point more clear at the introduction of the revised manuscript.

We agree with your point that both pharmaceutical and non-pharmaceutical approaches can be seen as interventions on human communities. We replaced the phrase "interventions on human communities" with your suggested term, "behavioral interventions" to make the distinction accurate.

RC: ***The first line second paragraph in the introduction talks about social distancing and the next line mentions vaccination as an example. As you mentioned earlier in the introduction, vaccination is not a social distancing intervention.***

AR: Thanks for pointing this out! While "getting vaccinated" (not developing the vaccine) is considered as a non-pharmaceutical effort as it also affects the rate of the spread of the disease (please see the answer to the previous question), it is technically not a social distancing approach. We changed the referred paragraph accordingly.

RC: ***While creating a family pattern, individual demographic distributions are assigned independently. While this is not an unreasonable assumption, in reality, demographics of people in the same family aren't independent of each other and it might be good to acknowledge this. The same argument can be made for the activities of family members.***

AR: Such distributions (even conditionally) are all given as input to the simulator in an easy-to-change manner. One can easily substitute the distributions of the locations by the conditional distribution of the location given to the household. However, the assumption you mentioned is a more realizable setting and is a better model for the simulation. We take into account such details in the repository.

RC: ***4. Page 3, "Interaction model: The transmission of the disease is through an underlying graph that determines the structure of the population.". Actually, the structure of the population (i.e., demographics, communities, and activities/mobility patterns) determines the underlying graph over which the disease transmission occurs.***

AR: Thanks for pointing this out! That is true as the population generation phase that creates the underlying graph is informed by the attributes from the population. We corrected this in the revised manuscript by changing "determines" to "is determined" to reverse the causal direction.

RC: ***5. I think some of the events names may be a bit confusing. For example, the paper mentions "Incubation***

event occurs when the incubation period is over and indicates a transition from the incubation period to the illness period during which the patient becomes infectious.". I think as this marks start of infectious period it should not be called an incubation event, but may be an infection event. Similarly, as the paper mentions "Infection Event occurs when the infection of an individual ends.". As this marks recovery (or death), may be this should be called Removed event.

AR: This was addressed by providing more explanations about the events as we thought the confusion comes from the insufficient description of the events. The events are queued and triggered at different times, so the naming makes more sense with the additional explanations that we provided in Section 3.2.

RC: ***6. Section 3.3.4 mentions consistency in results for different clock periods (temporal resolutions). It would be good to show this experimentally.***

AR: A paragraph + corresponding plots related to this matter is added to the revised manuscript in Figure 9 of Section 4.2.1.

RC: ***7. Many of the figures (e.g., Figure 9) show confidence intervals. It would be good to specify exactly what levels of confidence intervals are shown (e.g.,*** 95% ***confidence intervals).***

AR: We would like to emphasize that the error bands of the plots account for the stochastic nature of Pyfectious realized in several executions. In other words, the error bands are plotted along with the average to capture both stochasticity and the trend of the graphs in simulation time. As mentioned earlier, Pyfectious is stochastic at every level and this makes the whole population, disease, and daily schedule large random variables formed by many constituent components. Hence, every simulated path can be seen as a realization of the stochastic process (time evolution) of a measured property of the interactions among these random variables.

RC: ***8. Finding optimal policy could be computationally expensive. It would be good to mention run time for optimal policy estimation for the example in Figure 15 and Table 7.***

AR: It was added to the descriptions in Table 7. However, since we reduced the town size for that part (as explained in the descriptions), the execution time is not in the same range as in the other experiments in the paper.

RC: ***Minor edits:***
1. Page 2, "Population model: We enumerate various aspects of generating the society by creating the individuals." –> "Population model: We enumerate various aspects of generating a society by creating a set of individuals."

AR: Thanks!, Corrected!

RC: ***2. Page 9, section 3.1.1 Personal attributes, "disease, blood pressure, and etc." –> "disease, blood pressure, etc."***

AR: Thanks! Corrected!

RC: ***3. Page 9, section 3.1.2, Should "Its attributes are then are sampled from*** ϕ_i ***independent of other members." be "Its attributes are then sampled from*** φ_i***, independent of other members."?***

AR: Thanks! Corrected!

RC: ***4. I think, Algorithm 1 should take*** $(\phi_1, \phi_2, \dots \phi_n)$ ***and*** $(\pi_1, \pi_2, \dots \pi_n)$ ***as input instead of*** $(\varphi_1, \varphi_2, \dots, \varphi_n)$***. Also, should*** φ_j ***in line 5 be*** ϕ_j***?***

AR: The family patterns ϕ_1's are realized from $(\phi_1, \phi_2, \dots \phi_n)$ distributions, i.e. $(\varphi_1, \varphi_2, \dots \varphi_n)$. As the sampling

occurs within this algorithm, the distributions are given as input to it instead of the realized patterns.

We added $(\pi_1, \pi_2, \dots \pi_n)$ as input to the pseudocode algorithm in the revised manuscript.

RC: ***5. Page 12, should there be infinity symbol in the last line of "Special case of profession assignment" section?***

AR: Addressed! Thanks!

RC: ***6. Figure 5 caption, "from" is mentioned twice in the last line.***

AR: Thanks! Corrected!

RC: ***7. Figure 11b, it seems like strategy A and B are swapped between the plot and caption. Strategy B (not A as mentioned in the caption) is the one that enforces quarantine after 20 days and lifts it after 20 days.***

AR: We appreciate noticing this mismatch between labels and description. We resolved this.

RC: ***8. Figure 12a, it seems like strategies C, D, B, and A (not A, B, C, and D as mentioned in the caption) enforce a quarantine after 40, 60, 100, and 200 days after the outbreak, respectively.***

AR: We appreciate noticing this mismatch between labels and description. It is resolved.

RC: ***9. Page 34, I could not find https://github.com/amehrjou/Pyfectious/tree/master/example but was able to reach the Google Colaboratory Notebook from the project page on Github. Please update the link in the paper accordingly.***

AR: This issue was resolved and the link is replaced by a link to our Colaboratory notebook in the Appendix.

References

[1] Mark A Beaumont, Wenyang Zhang, and David J Balding. Approximate bayesian computation in population genetics. *Genetics*, 162(4):2025–2035, 2002.

[2] Jan C Thiele, Winfried Kurth, and Volker Grimm. Facilitating parameter estimation and sensitivity analysis of agent-based models: A cookbook using netlogo and r. *Journal of Artificial Societies and Social Simulation*, 17(3):11, 2014.

[3] Mickael Binois, Robert B Gramacy, and Mike Ludkovski. Practical heteroscedastic gaussian process modeling for large simulation experiments. *Journal of Computational and Graphical Statistics*, 27(4):808–821, 2018.

[4] Sen Pei, Flaviano Morone, Fredrik Liljeros, Hernán Makse, and Jeffrey L Shaman. Inference and control of the nosocomial transmission of methicillin-resistant staphylococcus aureus. *Elife*, 7:e40977, 2018.

[5] Peter Jagers. A plea for stochastic population dynamics. *Journal of mathematical biology*, 60(5):761–764, 2010.

[6] James C Cajka, Philip C Cooley, and William D Wheaton. Attribute assignment to a synthetic population in support of agent-based disease modeling. *Methods report (RTI Press)*, 19(1009):1, 2010.

[7] Shannon Gallagher, Lee F Richardson, Samuel L Ventura, and William F Eddy. Spew: synthetic populations and ecosystems of the world. *Journal of Computational and Graphical Statistics*, 27(4):773–784, 2018.

[8] Phillip Stroud, Sara Del Valle, Stephen Sydoriak, Jane Riese, and Susan Mniszewski. Spatial dynamics of pandemic influenza in a massive artificial society. *Journal of Artificial Societies and Social Simulation*, 10(4):9, 2007.

[9] Samuel M Jenness, Steven M Goodreau, and Martina Morris. Epimodel: an r package for mathematical modeling of infectious disease over networks. *Journal of statistical software*, 84, 2018.

[10] Lars Lorch, H Kremer, W Trouleau, S Tsirtsis, A Szanto, B Schölkopf, and M Gomez-Rodriguez. Quantifying the effects of contact tracing, testing, and containment measures in the presence of infection hotspots. 2020.

[11] Per Block, Marion Hoffman, Isabel J Raabe, Jennifer Beam Dowd, Charles Rahal, Ridhi Kashyap, and Melinda C Mills. Social network-based distancing strategies to flatten the covid-19 curve in a post-lockdown world. *Nature Human Behaviour*, pages 1–9, 2020.

[12] Mohammad Akbarpour, Cody Cook, Aude Marzuoli, Simon Mongey, Abhishek Nagaraj, Matteo Saccarola, Pietro Tebaldi, Shoshana Vasserman, and Hanbin Yang. Socioeconomic network heterogeneity and pandemic policy response. Technical report, National Bureau of Economic Research, 2020.

[13] Michael J North, Nicholson T Collier, Jonathan Ozik, Eric R Tatara, Charles M Macal, Mark Bragen, and Pam Sydelko. Complex adaptive systems modeling with repast simphony. *Complex adaptive systems modeling*, 1(1):1–26, 2013.
