# Supplementary material for: Pyfectious: An individual-level simulator to discover optimal containment policies for epidemic diseases
Source: PLoS Comput Biol. 2023 Jan 23;19(1):e1010799. doi: 10.1371/journal.pcbi.1010799 (PMC9894541; doi:10.1371/journal.pcbi.1010799)
Supplement: Attachment — Submitted filename: Response_second_reviewing_round.pdf [file pcbi.1010799.s006.pdf]

Authors' Response to Reviews of

Pyfectious: An individual-level simulator to discover optimal containment policies for epidemic diseases

A. Mehrjou, A. Soleymani, A. Abyaneh, S. Bhatt, B. Schölkopf, S. Bauer
Submitted to the journal of PLOS Computational Biology

RC: ***Reviewers' Comment***, AR: Authors' Response, ☐ Manuscript Text

We thank all reviewers for recognizing our work as a valuable contribution and appreciating our response to the first round of reviews. The valuable feedbacks in the first round helped us improve the presentation of our work significantly. In this letter, we tried to answer the remaining concerns of the respected reviewers and edited the manuscript accordingly. We would like to point out a few general points here: (1) The numbering of equations, sections, and figures are based on the new (revised) manuscript unless otherwise stated. (2) While all edited and added parts in the first round of the reviews were colored red, the new changes are colored blue in this round to be distinguishable from previous changes.

1. Reviewer #2

RC: ***The authors have made a substantial effort to address reviewer comments. The accessibility of this software make it a valuable entry point for many. The ability to specify the configuration of populations using JSON specifications that capture properties of real world populations is likely to occupy a space for computational epidemiology similar to other powerful yet accessible python libraries.***

AR: We would like to thank the reviewer for the positive feedback and for recognizing the major design principles of Pyfectious such as the accessibility and the straightforward way of defining the population configuration.

2. Reviewer #4

RC: ***The paper describes Pyfectious, a Python package that allows simulation of a disease outbreak at the individual level. It allows creating a probabilistic model of population, simulation of a wide range of policies, and finding optimal control policies using a reinforcement learning framework. The paper provides detailed description of the system and has addressed previous reviewer comments.***

AR: We sincerely thank you for describing the contributions of our work and especially for your feedback and questions that we try to answer in this letter.

RC: ***The first paragraph on Page 25 (The case of Multi-Resolution Simulation) states following: "Results in low-resolution (T=320 mins) is asymptotically consistent with very-high resolution regimes (T=40 mins)." It is not clear what they mean by "asymptotically consistent" here. There is a large difference in the number of infections over time (as well as peak and total number of infections) between T=40 mins and T=320 mins regimes, which could lead to different conclusions when the clock period is changed. My understanding is that one of the claims of the paper is that Pyfectious can provide similar results by changing clock period (or adjusting computational requirements) but some of the important results/numbers do not seem to hold when clock period is adjusted.***

AR:

Thank you for bringing this ambiguity to our attention. We should have used better wording to describe the intended behavior than "asymptotically consistent" as this phrase has a certain meaning in statistics and probability theory. We hereby describe what we meant by this phrase and why it is a useful desirable feature of Pyfectious. Moreover, we ran new experiments to support the following explanation whose results are shown the new Figure 9 of the revised manuscript.

Limited computational resources are often the bottleneck of simulation-based studies. That is, independent researchers or smaller institutions with access to only regular personal computers cannot derive meaningful conclusions from computation-heavy simulators. Hence, for a simulator to be accessible to a wider audience, not only resourceful institutions, it must be able to produce meaningful and useful results within a reasonable range of computational resources. Pyfectious introduces temporal resolution as a handle that trades off computation vs simulation details. The essence of this trade-off is that "some of the important results" of the simulation remain "almost invariant" under "reasonable reduction of the temporal resolution" which corresponds to a reduction in the required computation. In the following, we describe each of the quoted terms to shed more light on the intended meaning:

- Some of the important results: Reducing the temporal resolution obviously eliminates some of the details from the simulation results. However, we observed empirically that reducing the temporal resolution to a *reasonable degree* would not lead to a significant change in the major characteristics of the results such as the number of deaths or the overall shape of the curve of active cases over time (See Figure 9 (a) of the revised manuscript). These characteristics are of prime importance for policy search either by an RL agent or by human policymakers.
- Almost invariant: It is clear that the aforementioned characteristics do not remain completely unchanged as a result of reducing the temporal resolution. However, empirical results show that the resultant change is not so significant that changes the qualitative characteristics of the simulations and affects the downstream policy search algorithms.
- Reasonable reduction of the temporal resolution: The above two items hold if the temporal resolution of the simulator is not radically reduced. This is expected as one cannot expect to gain the exact same results by a very low temporal resolution (See Figure 9 (b) of the revised manuscript). We empirically showed that a reduction by almost 300% of temporal resolution does not change the main conclusions derived from the simulation results.

In summary, the takeaway message of the first paragraph on Page 25 and the corresponding figure is that although achieving the full-detailed simulation results requires high temporal resolution and consequently exceedingly high computational resources, Pyfectious allows reducing the temporal resolution to a reasonable fraction to save computation and derive almost the same conclusions from the simulations. To gain a better insight, we re-ran the experiments of Figure 9 again with many more resolution values to observe the transition from invariance to non-invariance results when the resolution drops below a certain threshold. Figure 9 (a) shows the simulation curves for a range of resolution values which shows the curves have maintained their key qualitative characteristics. However, Figure 9 (b) shows a wider range of resolutions that cause radical changes in the simulation curves which consequently affects the conclusions made from these curves.

Notice that these insights have only been gained empirically. Deriving a theoretical lower bound for the temporal resolution that preserves certain characteristics of the simulation results does not seem straightforward due to the involvement of multiple components in the simulation and has not been done in this work.

We edited the corresponding part of the text according to the above description in the revised manuscript and also changed Figure 9 with complementary experimental results.

RC: ***The last line in the first paragraph of the introduction seems a bit off. May be something like following could be more suitable: "Considering the vaccine policy as behavioral intervention allows investigation of situations where there is a shortage of vaccine supply or the population is not fully vaccinated along with other behavioral interventions such as social distancing."***

AR: Thanks for the recommended change. We replace that sentence with the suggested alternative in the revised manuscript.

RC: ***The second paragraph on Page 3 states "We tried to compare with a diverse set of highly-respected simulators in terms of their attitude and simulation methods in different pieces of literature, ..". It is not clear what attitude refers to here.***

AR: By attitude, we refer to the purpose of the simulators, i.e. the problems they are designed to solve. For example, [1] follows socioeconomic goals whereas [2] concerns resource management in healthcare domains. In the revised manuscript, we replaced *attitude* with *goal* followed by an explanatory sentence to elaborate more on what is intended.

RC: ***Page 3, last paragraph, a reference is missing (??).***

AR: Thanks for catching this! The reference points to the software manual at the end of the manuscript. We corrected the reference in the revised manuscript.

RC: ***Page 4, interaction model: "The transmission of the disease is through an underlying graph that is determined the structure of the population." –> "The transmission of the disease is through an underlying graph that is determined by the structure of the population."***

AR: Thanks for noticing the dropped word! We corrected it in the revised manuscript.

RC: ***Page 5, first paragraph: "Pyfectious allows a much richer space of possible policies, even including probabilistic and conditional control which also is not suffering from interpretation ambiguities." –> "Pyfectious allows a much richer space of possible policies, including probabilistic and conditional control which do not suffer from interpretation ambiguities."***

AR: Thanks! We replaced the above-mentioned sentence with the suggested alternative in the revised manuscript.

RC: ***Page 5, Policy discovery, last sentence: "Similar way, control theory… " –> "Similarly, control theory..."***

AR: Thanks! We corrected the above sentence in the revised manuscript.

RC: ***Page 9: "Infection Event the event is queued once" –> "Infection Event is queued once"***

AR: Thanks! The extra word was removed in the revised manuscript.

RC: ***Page 9, last paragraph and Page 14, first paragraph in "Propagation of the infection" section refer to Section 2.2.1 and line 10. Line 10 from where (which algorithm)?***

AR: We appreciate your acute attention! The references are fixed!

References

[1] Mohammad Akbarpour, Cody Cook, Aude Marzuoli, Simon Mongey, Abhishek Nagaraj, Matteo Saccarola, Pietro Tebaldi, Shoshana Vasserman, and Hanbin Yang. Socioeconomic network heterogeneity and pandemic policy response. Technical report, National Bureau of Economic Research, 2020.

[2] Levent Eriskin, Mumtaz Karatas, and Yu-Jun Zheng. A robust multi-objective model for healthcare resource management and location planning during pandemics. *Annals of Operations Research*, pages 1–48, 2022.
